# Supplementary material for: The effect of health insurance coverage on antenatal care utilization in Cambodia: A secondary analysis of Cambodia Demographic and Health Survey 2021–2022
Source: PLOS Glob Public Health. 2024 Nov 4;4(11):e0002954. doi: 10.1371/journal.pgph.0002954 (PMC11534221; doi:10.1371/journal.pgph.0002954)
Supplement: Attachment — Submitted filename: PGPH-D-24-00222_MN_1.pdf [file pgph.0002954.s002.pdf]

PLOS Global Public Health

Health insurance coverage and antenatal care utilization in Cambodia: Analysis of Cambodia Demographic and Health Survey 2021-22

--Manuscript Draft--

Manuscript Number:	PGPH-D-24-00222
Article Type:	Research Article
Full Title:	Health insurance coverage and antenatal care utilization in Cambodia: Analysis of Cambodia Demographic and Health Survey 2021-22
Short Title:	Health insurance coverage and antenatal care utilization in Cambodia
Corresponding Author:	Samnang Um, MSc<br>NIPH: National Institute of Public Health<br>Phnom Penh, Cambodia CAMBODIA
Order of Authors:	Samnang Um, MSc
	Channnarong Phan, MSc
	Khun Veha, MD
	Soklim Pay, MPH
	Darapheak Chau, MD,PhD
Keywords:	Key work: Health insurance; Antenatal care utilization; Pregnant women; Cambodia
Abstract:	Health insurance is essential in reducing or eliminating the financial constraint to accessing maternal health services caused by out-of-pocket payments. It has a beneficial effect in minimizing the number of maternal and child mortality. We examined the impact of health insurance coverage and antenatal care (ANC) utilization. We used data from the 2021-22 Cambodia Demographic and Health Surveys (CDHS). A total of 3,162 weighted women who gave birth within two years were included in the study. The association between health insurance coverage and pregnant women who attended at least four antenatal care visits during their pregnancy was evaluated using multiple logistic regression analysis. About 24.9% of women had health insurance coverage during 2021-22. Most (86.1%) of women attended at least 4 ANC visits. Almost 91.6% of women participating in four or more ANC visits were covered by health insurance. Having health insurance and attending four or more ANC visits were statistically significant adjusted odds ratio (AOR = 1.6; 95% CI: 1.1–2.4). Other factors significantly associated with attendance of four or more ANC visits include women with higher education (AOR = 3.1; 95% CI: 1.2–7.7), secondary education (AOR = 2.3; 95% CI: 1.5–3.5), richest households (AOR = 3.2; 95% CI: 1.5-6.8), and richer households (AOR = 1.9;95% CI: 1.2-2.8). Pregnant women with health insurance were more likely to attend at least four ANC visits. Thus, providing health insurance may be essential to improving women's access to maternal health services in Cambodia.
Additional Information:	
Question	Response
Financial Disclosure<br><br>Enter a financial disclosure statement that describes the sources of funding for the work included in this submission and the role the funder(s) played. This includes grants and any commercial funding of the work or authors.	The authors received no specific funding for this work.

Powered by Editorial Manager® and ProduXion Manager® from Aries Systems Corporation

This statement will be typeset if the manuscript is accepted for publication.<br><br>*Please review the submission guidelines and the instructions link below for detailed requirements and guidance.*	
**Competing Interests**<br><br>On behalf of all authors, disclose any competing interests that could be perceived to bias this work.<br><br>This statement will be typeset if the manuscript is accepted for publication.<br><br>*Please review the instructions link below and PLOS Global Public Health's competing interests policy to determine what information must be disclosed at submission.*	The authors have declared<br>that no competing interests exist.
**Data Availability**<br><br>Before publication, Authors are required to make fully available and without restriction all data underlying their findings. Please see our PLOS Data Policy page for detailed information on this policy.<br><br>A **Data Availability Statement**, detailing where the data can be accessed, is required at first submission. Insert your Data Availability Statement in the box below.<br><br>Please see the data reporting section of our submission guidelines for instructions on what you need to include in your Data Availability Statement.	The Cambodia Demographic and Health Survey data are publicly available from the website: (URL: https://www.dhsprogram.com/data/dataset_admin).

This statement will be typeset if the manuscript is accepted for publication.<br><br>*PLOS allows rare exemptions to address legal and ethical concerns. If you have legal or ethical restrictions, please use the box below to detail these in full sentences for the Journal team to consider.*	

Powered by Editorial Manager® and ProduXion Manager® from Aries Systems Corporation

Manuscript

Click here to access/download;Manuscript;Health Insurance in Cambodia.docx

Health insurance coverage and antenatal care utilization in Cambodia: Analysis of Cambodia Demographic and Health Survey 2021-22

Samnang Um[1,2*], Channnarong Phan[1], Khun Veha[3], Soklim Pay[4], Darapheak Chau[1]

1- National Institute of Public Health, Cambodia
2- Faculty of Social Science and Humanities, Royal University of Phnom Penh, Cambodia
3- Department of Payment Certification of National Payment Certification Agency, Cambodia
4- Technical Department of National Pediatric Hospital, Cambodia

* umsamnang56@gmail.com

Abstract

Health insurance is essential in reducing or eliminating the financial constraint to accessing maternal health services caused by out-of-pocket payments. It has a beneficial effect in minimizing the number of maternal and child mortality. We examined the impact of health insurance coverage and antenatal care (ANC) utilization. We used data from the 2021-22 Cambodia Demographic and Health Surveys (CDHS). A total of 3,162 weighted women who gave birth within two years were included in the study. The association between health insurance coverage and pregnant women who attended at least four antenatal care visits during their pregnancy was evaluated using multiple logistic regression analysis. About 24.9% of women had health insurance coverage during 2021-22. Most (86.1%) of women attended at least 4 ANC visits. Almost 91.6% of women participating in four or more ANC visits were covered by health insurance. Having health insurance and attending four or more ANC visits were statistically significant adjusted odds ratio (AOR = 1.6; 95% CI: 1.1–2.4). Other factors significantly associated with attendance of four or more ANC visits include women with higher education (AOR = 3.1; 95% CI: 1.2–7.7), secondary education (AOR = 2.3; 95% CI: 1.5–3.5), richest households (AOR = 3.2; 95% CI: 1.5-6.8), and richer households (AOR = 1.9;95% CI: 1.2-2.8). Pregnant women with health insurance were more likely to attend at least four ANC visits. Thus, providing health insurance may be essential to improving women's access to maternal health services in Cambodia.

Key work: Health insurance, Antenatal care utilization, Pregnant women, Cambodia

Introduction

Cambodia's maternal mortality rate has significantly declined in the past decade. Data from the 2021-22 Cambodia Demographic and Health Surveys (CDHS) show that maternal mortality had declined dramatically, from 488 to 154 per 100,000 live births between 2000 and 2021-22 [1,2]. By 2030, the global maternal death ratio is expected to drop to less than 70 per 100,000 live births, according to Sustainable Development Goal (SDG) 3.1[3]. This achievement can be attributed to the country's concerted effort to increase women's access to maternal health services, particularly the initiative to increase institutional births [4]. Institutional births dramatically increased, from 19.3% to 98%, while the proportion of pregnant women attending four or more antenatal care (ANC) appointments increased considerably, from 9% to 86.1%, between 2000 and 2021 [5]. In several studies, women who had health insurance had higher rates of using maternal health treatments, such as timely ANC and attending four or more ANC visits [6-9].

In 2019, the total population of Cambodia was 15.55 million, with 17.8% living below the national poverty line [10]. Since 2016, Cambodia has been classified as a lower-middle-income country, with gross domestic product (GDP) per capita from 302 US dollars in 2000 to 1,625 US dollars in 2021 [11]. Also, current expenditures on health per capita significantly increased from 20 US dollars in 2000 to 116 US dollars in 2020 [12]. Globally, 50% of people cannot access essential health services, as the World Bank and World Health Organization (WHO) reported in 2017 [13].

Cambodian National Social Security Fund (NSSF) has provided health insurance coverage to formal sector workers [14]. And poor households are covered by the Health Equity Fund (HEF), the co-financing mechanism of the government and development partners [15]. By 2025, the government intends to expand the reach of the NSSF health insurance program to include the entire population [16]. Thus, nearly 70% of the population aged 15-49 reported not covered by health insurance [5]. Health insurance coverage is expected to provide financial risk protection and reduce disparities in access by facilitating greater uptake of maternal health services [13]. To our knowledge, limited published peer-reviewed studies assess the association between health insurance coverage and access to maternal health services among women of reproductive age in Cambodia using updated data. One prior study on health insurance coverage and its impact on maternal healthcare utilization in low- and middle-income countries utilized data from CDHS 2010

[8]. This study included all women and men aged 15-49 and pooled Demographic and Health Survey (DHS) data in 30 low-and middle-income countries (LMICs) [8]. An additional study aimed to assess levels of health insurance coverage in 30 LMICs and examines the impact of health insurance status on the use of maternal health care in eight countries spanning sub-Saharan Africa (Burundi et al., Namibia, and Rwanda), West Asia (Albania), and South and Southeast Asia (Cambodia and Indonesia) [8]. Given the lack of scholarship addressing this health concern among Cambodian women aged 15-49, we examined the effects of health insurance coverage and antenatal care (ANC) utilization among pregnant women in Cambodia. The findings will provide a broader perspective on levels of health insurance coverage and the impact of health insurance status on the use of maternal health care in Cambodia. Additionally, the study will enable policymakers to understand better health insurance coverage among the adult population in Cambodia and proffer suggestions for improving universal health coverage in Cambodia.

Material and Methods

Ethical statement

The CDHS 2021–22 is publicly available, with all personal identifiers of study participants removed. Permission to analyze the data was granted by registering with the DHS program website at (URL: https://dhsprogram.com/data/available-datasets.cfm). Written informed consent was obtained from the parent/guardian of each participant under 18 before data collection. The Cambodia National Ethics Committee for Human Health Research (NECHR) approved the data collection tools and procedures for CHDS 2022 for Health Research on 10 May 2021 (Ref # 83 NECHR) and ICF's Institutional Review Board (IRB) in Rockville, Maryland, USA.

Data source

We used data from the most recent CDHS (2021–2022), a household survey conducted every five years nationally representative of the population [5]. The two-stage stratified cluster sampling method collected the samples from all provinces. At the first stage, clusters, or enumeration areas (EAs), that represent the entire country (urban and rural), are randomly selected from the sampling

frame using probability proportional (PPS) to cluster size. In the second stage, a complete listing of households was selected from each cluster using an equal probability systematic sampling, and then interviews were conducted with women aged 15–49 years who were born in the five years preceding the survey in the complete list selected households [5].

In total, 19,496 women aged 15-49 who had given birth in the last five years were interviewed face-to-face, using the survey standard questionnaire to collect information from women on several health indicators such as maternal health care service utilization, maternal and child health, nutrition, and reproductive health services [5]. Overall, 15,046 women who had not given birth in the past two years were excluded. Data restriction resulted in women who had a live birth in the past two years in a final analytic sample of 3,292 women (3,162 weighted women).

Measurements

Outcome variable

This study's outcome was the number of ANC visits during the last pregnancy among women aged 15-59 years (coded as 0 = less than 4 ANC visits and 1 = four and more ANC visits) [17].

Independent variable

The primary independent variable is maternal health insurance coverage (yes vs. no), including public and private insurance. The confounding variables included maternal factors: Women's age in years (15-30 and 31-49), Marital status (married and not married), Birth order (1st, 2nd, 3rd, and 4th or more), Education (no education, primary, and secondary or higher), Occupation (not working, professional, sales, services, agricultural, and manual labor), Individual Household factors include the Household wealth index (poorest, poorest, medium, richer, and richest), Geographical regions (Plains, Tonle Sap, Coastal/Sea, and Mountains), and Place of Residence (rural vs. urban).

Statistical analysis

Analyses were performed using STATA V17 (StataCorp LLC). We applied for the CDHS sampling weight variable (v005/1,000,000). Then, we used the survey-specific STATA command "svy" for

descriptive and analytical. Women's socio-economic and demographic characteristics were described using weighted frequency and percentage distributions.

Bivariate analysis using Chi-square tests assessed the association between the variables of interest (maternal and individual household characteristics) and ANC use. All independent variables associated with ANC use at p-value ≤ 0.10 were included in the multiple logistic regression analysis to determine the independent factors related to ANC use [26]. Multicollinearity between independent variables was checked, including women's age, number of children ever born, education, wealth index, occupation, marital status, coverage health insurance, and place of residence. Result of the evaluating variance inflation factor (VIF) scores after fitting an Ordinary Least Squares regression model. The mean value of VIF was 1.53, which is less than the cutoff point [27].

Results

Characteristics of the study population

Table 1 describes the socio-economic and demographic characteristics of the 3,162 women aged 15–49 born in the last two years preceding the survey. The mean age was 22.2 years old (SD = 4.2 years); the age group of 15–29 years old accounted for 94.3%. The majority (95%) were currently married. More than 33.4% of women were giving in the first birth order. Half of the women completed at least secondary education, while 10.6% had no formal education. Only 6.5% of workers are professionals, and 31.2% are unemployed. Of the sample, 20.7% of women were from the poorest households, and 19.7% were from poorer families. Sixty-two percent of the women lived in rural areas. Only 786 (24.9%) women aged 15–49 had health insurance coverage. 86.1% of women attended at least 4 ANC visits during pregnancy

Table 1. Socio-economic and demographic characteristics of women aged 15-49 years with a birth in the last two years preceding the survey (N = 3,162 weighted)

Variables	Freq.	%
**Mean age at the time of birth (SD)**	22.2(4.2)	
15-29	2,982	94.3
30-49	180	5.7
**Marital status**		
Married	3,004	95.0
Not married	158	5.0
**Birth order**		
1st child	1055	33.4
2nd child	1197	37.9
3rd child or more	909	28.7
**Educational**		
No education	334	10.6
Primary	1253	39.6
Secondary	1361	43.0
Higher	214	6.8
**Occupation**		
None	986	31.2
Professional	205	6.5
Sales	579	18.3
Agricultural	445	14.1
Services	76	2.4
Manual labor	810	25.6
**Wealth index**		
Poorest	655	20.7
Poorer	623	19.7
Middle	626	19.8
Richer	683	21.6
Richest	574	18.2

**Residence**		
Urban	1202	38.0
Rural	1960	62.0
**Region**		
Plain	1532	48.5
Tonle Sap	996	31.5
Coastal	201	6.4
Plateau/Mountain	432	13.7
**Covered by health insurance**		
No	2376	75.1
Yes	786	24.9
**Number of ANC visits**		
< 4 ANC	440	13.9
$\geq$ 4 ANC	2722	**86.1**

Notes: *Survey weights are applied to obtain weighted percentages. ****Plains:*** *Phnom Penh, Kampong Cham, Tbong Khmum, Kandal, Prey Veng, Svay Rieng, and Takeo;* ***Tonle Sap:*** *Banteay Meanchey, Kampong Chhnang, Kampong Thom, Pursat, Siem Reap, Battambang, Pailin, and Otdar Meanchey;* ***Coastal/sea:*** *Kampot, Kep, Preah Sihanouk, and Koh Kong;* ***Mountains:*** *Kampong Speu, Kratie, Preah Vihear, Stung Treng, Mondul Kiri, and Ratanak Kiri.*

Maternal health insurance characteristics

The highest proportion of women having health insurance was among those aged 15-30 years (25.3.4%, p = 0.094); higher education (48.1%, p < 0.001); worked in professional jobs (59.0%), manual labor (52.5%) with p < 0.001); and women from the richer households (30.2%), richest households (32.2%) with p <0.001); and women in urban areas (35.9%, p <0.001); (**see Table 2)**.

Table 2. Socio-economic and demographic characteristics by the health insurance coverage of women aged 15-49 years with a birth in the last two years preceding the survey (**N = 3,162**)

	Covered by health insurance				
Variables	No (N=2,367)		Yes (N=786)		p-value
**Age at time of birth**	**Freq.**	%	**Freq.**	%	
15-30	2,229	74.7	753	25.3	0.094
31-49	147	81.7	33	18.3	
**Marital status**					
Married	2,260	75.2	744	24.8	0.757
Not married	116	73.4	42	26.6	
**Birth order**					
1st child	778	73.7	277	26.3	0.035
2nd or 3rd child	876	73.2	321	26.8	
4th child or more	722	79.4	188	20.7	
**Educational**					
No education	269	80.5	65	19.5	<0.001
Primary	950	75.8	304	24.3	
Secondary	1,046	76.9	315	23.1	

Higher	111	51.9	103	48.1	
**Occupation**					
None	892	90.5	93	9.4	<0.001
Professional	84	41.0	121	59.0	
Sales	514	88.8	65	11.2	
Agricultural	387	87.0	58	13.0	
Services	64	84.2	12	15.8	
Manual labor	385	47.5	425	52.5	
**Wealth index**					
Poorest	537	82.0	118	18.0	<0.001
Poorer	493	79.1	131	21.0	
Middle	479	76.5	147	23.5	
Richer	477	69.8	206	30.2	
Richest	389	67.8	185	32.2	
**Residence**					
Urban	770	64.1	432	35.9	<0.001
Rural	1,606	81.9	354	18.1	
**Region**					
Plain	1,119	73.0	413	27.0	0.019
Tonle Sap	781	78.4	216	21.7	
Coastal	167	83.1	34	16.9	
Plateau/Mountain	309.0	71.5	123.0	28.5	

***Notes:** Survey weights are applied to obtain weighted percentages. ***Plains:** Phnom Penh, Kampong Cham, Tbong Khmum, Kandal, Prey Veng, Svay Rieng, and Takeo; **Tonle Sap:** Banteay Meanchey, Kampong Chhnang, Kampong Thom, Pursat, Siem Reap, Battambang, Pailin, and Otdar Meanchey; **Coastal/sea:** Kampot, Kep, Preah Sihanouk, and Koh Kong; **Mountains:** Kampong Speu, Kratie, Preah Vihear, Stung Treng, Mondul Kiri, and Ratanak Kiri.*

Factors Associated with Four or more ANC visits in Chi-Square analysis

In bivariate analysis (**Table 3**), a higher proportion of women with health insurance coverage had a significant association with four or more ANC visits (91.6% vs. 84.2%, $p < 0.001$). Women aged 31–49 reported being more likely to attend four or more ANC visits (88.0% vs. 86.0%, $p < 0.001$). Also, married women reported four or more ANC visits than nonmarried women (86.6% vs. 76.2%, $p = 0.007$). Women with no education were less likely to attend four or more ANC visits than those with higher education (71.0% vs. 95.6%, $p < 0.001$). Four or more ANC visits were higher among women working in professional (95.3%) and service (96.4%), respectively, compared to unemployed women (84.3), with $p < 0.001$). Additionally, four or more ANC visits were higher among women from the richer and richest on the wealth index (94.8 and 89.4%, respectively), compared to the poorer and poorest (73.9 and 86.9%, respectively, with $p < 0.001$). Lastly, women living in urban areas reported higher four or more ANC visits than in rural areas (91.5 vs. 82.7%, $p < 0.001$).

Table 3. Maternal and household characteristics by women attending at least four antenatal care and delivery in a health facility **(N = 3,162)**

Variables	Number of ANC visits		p-value
	**Four or more**	**Less than four**	
	**n=2,722**	**n=440**	
	%	%	
**Covered by health insurance**			
No	84.2	15.8	<0.001
Yes	91.6	8.4	
**Age at time of birth**			
15-30	86.0	14.0	<0.001
31-49	88.0	12.0	
**Marital status**			
Married	86.6	13.4	0.007
Not married	76.2	23.8	
**Birth order**			
1st child	88.4	11.6	<0.001
2nd or 3rd child	88.6	11.4	
4th child or more	80.1	19.9	
**Educational**			
No education	71.0	29.0	<0.001
Primary	84.1	15.9	
Secondary	90.1	9.9	
Higher	95.6	4.4	
**Occupation**			
None	84.3	15.7	<0.001

Professional	95.3	4.7	
Sales	84.4	15.6	
Agricultural	79.0	21.0	
Services	96.4	3.6	
Manual labor	89.9	10.1	
**Wealth index**			
Poorest	73.9	26.1	<0.001
Poorer	86.9	13.1	
Middle	86.3	13.7	
Richer	89.4	10.6	
Richest	94.8	5.2	
**Residence**			
Urban	91.5	8.5	<0.001
Rural	82.7	17.3	
**Region**			
Plain	89.3	10.7	<0.001
Tonle Sap	86.6	13.4	
Coastal	89.0	11.0	
Plateau/Mountain	72.2	27.8	

Notes: *Survey weights are applied to obtain weighted percentages. ****Plains:*** *Phnom Penh, Kampong Cham, Tbong Khmum, Kandal, Prey Veng, Svay Rieng, and Takeo;* ***Tonle Sap:*** *Banteay Meanchey, Kampong Chhnang, Kampong Thom, Pursat, Siem Reap, Battambang, Pailin, and Otdar Meanchey;* ***Coastal/sea:*** *Kampot, Kep, Preah Sihanouk, and Koh Kong;* ***Mountains:*** *Kampong Speu, Kratie, Preah Vihear, Stung Treng, Mondul Kiri, and Ratanak Kiri.*

Association between health insurance and maternal Healthcare Services Utilization

Table 4 shows the results of the multiple logistic regression analysis of the association between health insurance coverage and maternal healthcare services utilization after controlling for the socio-demographic factors. Compared to women without health insurance, those with health insurance coverage were more likely to attend four or more ANC visits (AOR = 1.6, 95% CI: 1.1–2.4). Women with higher education (AOR = 3.1, 95% CI: 1.2–7.7), secondary education (AOR = 2.3, 95% CI: 1.5–3.5), and primary education (AOR = 1.7, 95% CI: 1.2–2.7) were more likely to have four or more ANC visits than women without any formal education. The odds of having four or more ANC visits were more significant for women from the wealthiest households than for those from the poorest households: richest households (AOR = 3.2; 95% CI: 1.5-6.8), richer households (AOR = 1.9; 95% CI: 1.2-2.8), and middle households (AOR = 1.5; 95% CI: 1.1-2.2). However, the odds of having four or more ANC visits were lower in unmarried women than in married women (AOR = 0.5; 95% CI: 0.3-0.8).

Table 4. Association between health insurance and four or more ANC visits in simple and multiple logistic regression model **(N = 3,162)**

Variables	Four or more ANC visits			
	Unadjusted		Adjusted	
	OR	95% CI	AOR	95% CI
**Covered by health insurance**				
No	1.0		1.0	
Yes	2.0***	(1.4-2.9)	**1.6***	**(1.1-2.4)**
**Age at time of birth**				
15-30	1.0		1.0	
31-49	1.2	(0.7-2.1)	1.1	(0.6-2.0)
**Marital status**				
Married	1.0		1.0	
Not married	0.5**	(0.3-0.8)	**0.5****	**(0.3-0.8)**
**Birth order**				
1st child	1.0		1.0	
2nd or 3rd child	1.0	(0.8-1.4)	1.1	(0.8-1.5)
4th child or more	0.5***	(0.4-0.7)	0.7*	(0.5-1.0)
**Educational**				
No education	1.0		1.0	
Primary	2.2***	(1.5-3.1)	**1.8****	**(1.2-2.7)**
Secondary	3.7***	(2.6-5.3)	**2.3*****	**(1.5-3.5)**
Higher	8.9***	(4.3-18.3)	**3.1***	**(1.2-7.7)**
**Occupation**				
None	1.0		1.0	
Professional	3.8***	(2.0-7.0)	1.4	(0.7-2.8)

Sales	1.0	(0.7-1.4)	0.7	(0.5-1.0)
Agricultural	0.7*	(0.5-1.0)	1.0	(0.7-1.4)
Services	4.9**	(1.8-13.5)	2.6	(0.9-7.2)
Manual labor	1.7**	(1.2-2.3)	1.3	(0.9-1.9)
**Wealth index**				
Poorest	1.0		1.0	
Poorer	2.4***	(1.7-3.2)	**1.7****	**(1.2-2.3)**
Middle	2.2***	(1.6-3.1)	**1.5***	**(1.1-2.2)**
Richer	3.0***	(2.1-4.2)	**1.9****	**(1.2-2.8)**
Richest	6.4***	(3.4-12.0)	**3.2****	**(1.5-6.8)**
**Residence**				
Urban	1.0		1.0	
Rural	0.4***	(0.3-0.6)	0.8	(0.5-1.1)
**Region**				
Plain	1.0		1.0	
Tonle Sap	0.8	(0.6-1.1)	1.2	(0.9-1.7)
Coastal	1.0	(0.6-1.5)	1.3	(0.8-2.1)
Plateau/Mountain	0.3***	(0.2-0.4)	0.5***	(0.3-0.6)

Ref = reference value

* $p < 0.05$, ** $p < 0.01$, *** $p < 0.001$

Notes: *Survey weights are applied to obtain weighted percentages. ****Plains:*** *Phnom Penh, Kampong Cham, Tbong Khmum, Kandal, Prey Veng, Svay Rieng, and Takeo;* ***Tonle Sap:*** *Banteay Meanchey, Kampong Chhnang, Kampong Thom, Pursat, Siem Reap, Battambang, Pailin, and Otdar Meanchey;* ***Coastal/sea:*** *Kampot, Kep, Preah Sihanouk, and Koh Kong;* ***Mountains:*** *Kampong Speu, Kratie, Preah Vihear, Stung Treng, Mondul Kiri, and Ratanak Kiri.*

Discussion

We analyzed the most recent 2021–22 CDHS data to examine the relationship between health insurance coverage and receiving four or more ANC visits during pregnancy. Overall, 24.9% of women reported having health insurance coverage among women of reproductive age who gave birth within two years of the survey. This finding is similar to lower-middle-income countries, where 27.3% of women had health insurance coverage [18]. This is higher than in low-income countries, where 7.9% of women have health insurance coverage [18]. However, lower than in upper-middle-income countries, 52.5% of women had health insurance coverage [18]. Since the formal launch of the Cambodia National Social Security Fund (NSSF) with the Health Insurance Scheme in 2008, the proportion of women with health insurance coverage has increased from 16% in 2014 to 22% in 2021–22 [5]. This proportion exponentially increased due to the Royal Government of Cambodia's implementation of the NSSF for all workers in the formal and informal sectors of the economy [10]. Moreover, it has plans to extend the healthcare benefits under the NSSF to the family members of the employees as well [16].

The proportion of women with health insurance coverage was found to be highest among women with higher levels of education and those from better households. This is in line with a meta-analysis of 48 studies conducted in 17 countries, which revealed a higher likelihood of health insurance participation in the wealthiest and most educated households [19]. Additionally, women with higher educational levels and wealth status consistently predicted health insurance ownership among women at reproductive age across five Sub-Saharan African countries [20]. Furthermore, we fund women with health insurance coverage, a lower proportion among women living in rural areas. Consistent with studies in Nigeria [9]. This suggests that the more vulnerable populations, such as the poor, rural areas, and least educated, should be reached with government interventions such as having health insurance coverage.

The proportion of pregnant women attending four or more antenatal care appointments has increased considerably from 9% to 86.1% between 2000 and 2021. This study found that women with health insurance coverage were 1.6 times more likely to attend four or more ANC visits during pregnancy. Previous studies documented the positive relationship between health insurance and the number of ANC visits among women of reproductive age [8,9,18]. Health insurance eliminates the financial barrier to accessing maternal health services caused by out-of-pocket payments. It has a beneficial effect in reducing the number of low-birth-weight babies born and child mortality [21,22]. The result is more equitable access to care, potentially improving maternal health outcomes [9,18]. The MoH has since raised the minimum standard for ANC visits during pregnancy to at least four trips [17,23]. The dramatically significant increase in the highest prevalence of four or more ANC visits was an effort by the Royal Government of Cambodia, which has strengthened health facilities across the country, particularly in rural areas, improved infrastructure, provided essential medical equipment and supplies, increased the number of midwives, expanded antenatal care, and provided more skilled medical practitioners at childbirth to ensure safe delivery practices. Furthermore, to encourage early and routine ANC visits, the government is offering pregnant women a monetary incentive of US$20 for each visit during a maximum of four ANC visits at any health facility with a contract with the National Social Security Fund (NSSF) [14,24].

This study found that increased education and household wealth index increased the likelihood of four or more ANC visits. Women's education and higher wealth index are more likely to attend four or more ANC visits. This aligns with previous evidence around socio-economic inequalities

in maternal health service utilization in Cambodia and South Asia [25,26]. This may be because the more educated the woman is, the more aware she is of the importance of ANC for her health and baby [27]. Additionally, education gives women the power to decide whether to seek medical attention and enables them to recognize warning signs of pregnancy complications. On the other hand, women from higher-income households were more likely to be able to cover the costs of care-seeking, including any related expenses and transportation [25,26]. Thus, in this study, women with higher education levels and household wealth indexes had the highest proportion of health insurance coverage.

This study has several strengths. First, it used the most recent women's data from the 2021–22 CDHS, an extensive representative national population-based household survey with a high response rate of 97%. Second, the recall bias has been minimized by limited analysis of women's most recent deliveries within the last two years preceding the survey [5]. Data were collected using validated survey methods and highly trained data collectors, contributing to improved data quality [28]. Third, the complex survey design and sampling weights in the analysis accounted for both descriptive and analytics methods that enabled us to generalize our findings to the population of WRA in Cambodia. Lastly, to our knowledge, this is the first study to report the association between health insurance coverage and ANC visits in Cambodia. After controlling sociodemographic factors, we found significant associations between health insurance coverage and several forums or more ANC visits. A key finding compares the association with other studies in Southeast Asia and globally.

Despite this, there are several limitations. First, this study was a household-based survey, so it did not address health institution factors of antenatal care utilization and service availability; hence, this study could not explore the quality of ANC services, though the quality of healthcare services plays a vital role in patient satisfaction and use. Second, the study's cross-sectional nature could not assist in the temporal relationship of variables. Therefore, further study should be conducted to identify factors related to health institutions. In addition, antenatal care utilization should be performed based on the new WHO guidelines at the national level [23].

Moreover, longitudinal studies that address comprehensive variables should be studied. Third, we excluded other factors, such as maternal complications and women's empowerment indicators, that could affect the use of maternal care. Lastly, CDHS did not assess a direct measure of maternal health literacy.

Conclusion

This is the first study to report the association between health insurance coverage and ANC visits in the healthcare setting in Cambodia. Cambodian pregnant women attend four or more antenatal care visits, which is slightly high. However, it still needs to be satisfactory. Health among coverage women of reproductive age in Cambodia is relatively low. Moreover, we found that women with health insurance, women with education, and being rich in the wealth quintile were strong predictors of women attending four or more ANC visits. There is a need to pay close attention to improving the uptake of health insurance among women of reproductive age, especially targeting women with no education, from low-income families, and women who reside in rural areas. Policymakers may need to prioritize women of reproductive age in designing and implementing health insurance programs to increase their uptake. This would provide financial risk protection, facilitate access to maternal health services, and possible attainment of Cambodia’s SDG 3 targets.

Acknowledgments

The authors would like to thank DHS-ICF, who approved the data used for this paper.

References

1. National Institute of Statistics, Directorate General for Health, and ICF International. Cambodia Demographic and Health Survey 2014. Phnom Penh, Cambodia, and Rockville, Maryland, USA: National

Institute of Statistics, Directorate General for Health, and ICF International. Available online: https://dhsprogram.com/pubs/pdf/fr312/fr312.pdf. 2015.
2. National Institute of Statistics PP, Cambodia, Ministry of Health Phnom Penh, Cambodia, The DHS Program, ICF, Rockville, Maryland, USA. Cambodia Demographic and Health Survey 2021–22. Phnom Penh, Cambodia, and Rockville, Maryland, USA: NIS, MoH, and ICF. 2022. Available from: https://dhsprogram.com/pubs/pdf/FR377/FR377.pdf.
3. The United Nations Development Programme. The Sustainable Development Goals by 2016-2030. Available from: https://www.undp.org/content/undp/en/home/sustainable-development-goals.html.
4. Acuin CS, Khor GL, Liabsuetrakul T, Achadi EL, Htay TT, Firestone R, et al. In southeast Asia, maternal, neonatal, and child health: towards greater regional collaboration. Lancet. 2011;377(9764):516-25. Epub 20110125. doi: 10.1016/s0140-6736(10)62049-1. PubMed PMID: 21269675; PubMed Central PMCID: PMCPMC7159081.
5. National Institute of Statistics (NIS) MoHM, and ICF. . Cambodia Demographic and Health Survey 2021–22 Final Report. Phnom Penh, Cambodia, and Rockville, Maryland, USA: NIS, MoH, and ICF. 2023. Available from: https://dhsprogram.com/pubs/pdf/PR136/PR136.pdf.
6. Merga BT, Raru TB, Deressa A, Regassa LD, Gamachu M, Negash B, et al. The effect of health insurance coverage on antenatal care utilizations in Ethiopia: evidence from national survey. Front Health Serv. 2023;3:1101164. Epub 20231006. doi: 10.3389/frhs.2023.1101164. PubMed PMID: 37869683; PubMed Central PMCID: PMCPMC10587574.
7. Aboagye RG, Okyere J, Ahinkorah BO, Seidu AA, Zegeye B, Amu H, et al. Health insurance coverage and timely antenatal care attendance in sub-Saharan Africa. BMC Health Serv Res. 2022;22(1):181. Epub 20220211. doi: 10.1186/s12913-022-07601-6. PubMed PMID: 35148769; PubMed Central PMCID: PMCPMC8840787.
8. Wang W, Gheda Temsah, and Lindsay Mallick. . Health Insurance Coverage and Its Impact on Maternal Health Care Utilization in Low- and Middle-Income Countries. DHS Analytical Studies No. 45. Rockville, Maryland, USA: ICF International. . 2014.
9. Ahuru RR, Omon IJ, Nzoputam CI, Ekomoezor E. Health insurance ownership and maternal health service uptake among Nigerian women. HEALTH. 2021;12.
10. National Institute of Statistics. Ministry of Planning. General population census of the Kingdom of Cambodia 2019. Available from: https://www.nis.gov.kh/nis/Census2019/Final%20General%20Population%20Census%202019-English.pdf.
11. The World Bank In Cambodia. Overview 2019. Available from: https://www.worldbank.org/en/country/cambodia/overview.
12. World Health Organization (WHO). Global Health Expenditure Database 2019. Available from: https://apps.who.int/nha/database/PHC_Country_profile/Index/en.
13. World Health Organization (WHO); World Bank and WHO. Half the world lacks access to essential health services, 100 million still pushed into extreme poverty because of health expenses 2017. 2017. Available from: http://www.who.int/mediacentre/news/releases/2017/half-lacks-access/en/.
14. National Social Security Fund (NSSF). Health Insurance Scheme 2016. Available from: https://www.nssf.gov.kh/health-care-scheme-2/.
15. Nakamura H, Amimo F, Yi S, Tuot S, Yoshida T, Tobe M, et al. Implementing a sustainable health insurance system in Cambodia: a study protocol for developing and validating an efficient household income-level assessment model for equitable premium collection. Int J Equity Health. 2020;19(1):17. Epub 20200131. doi: 10.1186/s12939-020-1126-8. PubMed PMID: 32005237; PubMed Central PMCID: PMCPMC6995079.
16. The Royal Government of Cambodia. National Social Protection Policy Framework 2016–2025. Phnom Penh: The Council of Ministers; 2017.
17. Ministry of Health. Guideline for implementation the service package of Antenatal Care Delivery and Post natal Care 2019 [updated 2019]. 3:[Available from: https://niph.org.kh/niph/uploads/library/pdf/GL220_NMCHC_anc_pnc_package_eng.pdf.

18. Hooley B, Afriyie DO, Fink G, Tediosi F. Health insurance coverage in low-income and middle-income countries: progress made to date and related changes in private and public health expenditure. BMJ Glob Health. 2022;7(5). doi: 10.1136/bmjgh-2022-008722. PubMed PMID: 35537761; PubMed Central PMCID: PMCPMC9092126.
19. Osei Afriyie D, Krasniq B, Hooley B, Tediosi F, Fink G. Equity in health insurance schemes enrollment in low and middle-income countries: A systematic review and meta-analysis. Int J Equity Health. 2022;21(1):21. Epub 20220212. doi: 10.1186/s12939-021-01608-x. PubMed PMID: 35151323; PubMed Central PMCID: PMCPMC8841076.
20. Shao L, Wang Y, Wang X, Ji L, Huang R. Factors associated with health insurance ownership among women of reproductive age: A multicountry study in sub-Saharan Africa. PLoS One. 2022;17(4):e0264377. Epub 20220412. doi: 10.1371/journal.pone.0264377. PubMed PMID: 35413063; PubMed Central PMCID: PMCPMC9004737.
21. Likka MH, Handalo DM, Weldsilase YA, Sinkie SO. The effect of community-based health insurance schemes on utilization of healthcare services in low- and middle-income countries: a systematic review protocol of quantitative evidence. JBI Database System Rev Implement Rep. 2018;16(3):653-61. doi: 10.11124/jbisrir-2017-003381. PubMed PMID: 29521866.
22. Imo CK, De Wet-Billings N, Isiugo-Abanihe UC. The impact of maternal health insurance coverage and adequate healthcare services utilisation on the risk of under-five mortality in Nigeria: a cross-sectional study. Arch Public Health. 2022;80(1):206. Epub 20220913. doi: 10.1186/s13690-022-00968-2. PubMed PMID: 36100949; PubMed Central PMCID: PMCPMC9472384.
23. WHO Guidelines Approved by the Guidelines Review Committee. WHO Recommendations on Antenatal Care for a Positive Pregnancy Experience. Geneva: World Health Organization

Copyright © World Health Organization 2016.; 2016.
24. Ministry of Information. Government Raises Allowances For Mothers And Children. Available from: https://www.information.gov.kh/articles/108509.
25. Chham S, Radovich E, Buffel V, Ir P, Wouters E. Determinants of the continuum of maternal health care in Cambodia: an analysis of the Cambodia demographic health survey 2014. BMC Pregnancy Childbirth. 2021;21(1):410. Epub 20210602. doi: 10.1186/s12884-021-03890-7. PubMed PMID: 34078318; PubMed Central PMCID: PMCPMC8170811.
26. Lee HY, Oh J, Kim R, Subramanian SV. Long-term trend in socio-economic inequalities and geographic variation in the utilization of antenatal care service in India between 1998 and 2015. Health Serv Res. 2020;55(3):419-31. Epub 20200304. doi: 10.1111/1475-6773.13277. PubMed PMID: 32133652; PubMed Central PMCID: PMCPMC7240766.
27. Thakkar N, Alam P, Saxena D. Factors associated with underutilization of antenatal care in India: Results from 2019-2021 National Family Health Survey. PLoS One. 2023;18(5):e0285454. Epub 20230508. doi: 10.1371/journal.pone.0285454. PubMed PMID: 37155665; PubMed Central PMCID: PMCPMC10166529.
28. Allen C, Fleuret J, Ahmed J, editors. Data quality in demographic and health surveys that used long and short questionnaires2020: ICF.

Click here to access/download
Supporting Information
PLOSOne_Human_Subjects_Research_Checklist.docx
