# Supplementary material for: The effect of health insurance coverage on antenatal care utilization in Cambodia: A secondary analysis of Cambodia Demographic and Health Survey 2021–2022
Source: PLOS Glob Public Health. 2024 Nov 4;4(11):e0002954. doi: 10.1371/journal.pgph.0002954 (PMC11534221; doi:10.1371/journal.pgph.0002954)
Supplement: Attachment — Submitted filename: PGPH-D-24-00222_R1_reviewer_MN.pdf [file pgph.0002954.s004.pdf]

PLOS Global Public Health

The effect of health insurance coverage on antenatal care utilization in Cambodia: A secondary analysis of Cambodia Demographic and Health Survey 2021-2022

--Manuscript Draft--

Manuscript Number:	PGPH-D-24-00222R1
Article Type:	Research Article
Full Title:	The effect of health insurance coverage on antenatal care utilization in Cambodia: A secondary analysis of Cambodia Demographic and Health Survey 2021-2022
Short Title:	The effect of health insurance coverage on antenatal care utilization in Cambodia
Corresponding Author:	Samnang Um, MSc<br>NIPH: National Institute of Public Health<br>Phnom Penh, Cambodia CAMBODIA
Order of Authors:	Samnang Um, MSc
	Channnarong Phan, MSc
	Khun Veha, MD
	Soklim Pay, MPH
	Darapheak Chau, MD,PhD
Keywords:	Key work: Health insurance; Antenatal care utilization; Pregnant women; Cambodia
Abstract:	Health insurance is essential in reducing or eliminating the financial constraint to accessing maternal health services caused by out-of-pocket payments. Also, it has a beneficial effect in minimizing the number of maternal and child mortality. However, limited studies in Cambodia examined the association between health insurance coverage on antenatal care (ANC) utilization. Therefore, this study examined the effect of health insurance coverage on ANC utilization in Cambodia. The study utilized women's data from the 2021-2022 Cambodia Demographic and Health Surveys (CDHS). A total of 3,162 weighted women who gave birth within two years were included in the study. Multiple logistic regression analysis was explored to assess the association between health insurance coverage on women who attended four or more ANC visits. About 24.9% of the women had health insurance coverage during 2021-2022. Most (86.1%) of women attended at least four ANC visits. Women with health insurance coverage were statistically significantly associated with attending four or more ANC visits with an adjusted odds ratio (AOR = 1.6; 95% CI: 1.1–2.4). Other covariates significantly associated with women who attended four or more ANC visits include women with higher education (AOR = 3.1; 95% CI: 1.2–7.7), secondary education (AOR = 2.3; 95% CI: 1.5–3.5), richest households (AOR = 3.2; 95% CI: 1.5-6.8), and richer households (AOR = 1.9;95% CI: 1.2-2.8). Pregnant women with health insurance coverage, who had completed at least secondary education and had a better wealth index were more likely to attend at least four ANC visits. Thus, improving health insurance coverage, economics, and education may be essential to improving women's access to attended ANC utilization services in Cambodia.
Additional Information:	
**Question**	**Response**
**Financial Disclosure**<br><br>Enter a financial disclosure statement that describes the sources of funding for the work included in this submission and the role the funder(s) played. This includes grants and any commercial funding of the	The authors received no specific funding for this work.

Powered by Editorial Manager® and ProduXion Manager® from Aries Systems Corporation

work or authors.<br><br>This statement will be typeset if the manuscript is accepted for publication.<br><br>*Please review the submission guidelines and the instructions link below for detailed requirements and guidance.*	
**Competing Interests**<br><br>On behalf of all authors, disclose any competing interests that could be perceived to bias this work.<br><br>This statement will be typeset if the manuscript is accepted for publication.<br><br>*Please review the instructions link below and PLOS Global Public Health's competing interests policy to determine what information must be disclosed at submission.*	The authors have declared<br>that no competing interests exist.
**Data Availability**<br><br>Before publication, Authors are required to make fully available and without restriction all data underlying their findings. Please see our PLOS Data Policy page for detailed information on this policy.<br><br>A **Data Availability Statement**, detailing where the data can be accessed, is required at first submission. Insert your Data Availability Statement in the box below.<br><br>Please see the data reporting section of our submission guidelines for instructions on what you need to include in your Data Availability Statement.	The Cambodia Demographic and Health Survey data are publicly available from the website: (URL: https://www.dhsprogram.com/data/dataset_admin).

This statement will be typeset if the manuscript is accepted for publication.<br><br>*PLOS allows rare exemptions to address legal and ethical concerns. If you have legal or ethical restrictions, please use the box below to detail these in full sentences for the Journal team to consider.*	

Manuscript Click here to access/download;Manuscript;Manuscript.docx

The effect of health insurance coverage on antenatal care utilization in Cambodia: A secondary analysis of Cambodia Demographic and Health Survey 2021-2022

Samnang Um[1,2*], Channnarong Phan[1], Khun Veha[3], Soklim Pay[4], Darapheak Chau[1]

1- National Institute of Public Health, Cambodia
2- Faculty of Social Science and Humanities, Royal University of Phnom Penh, Cambodia
3- Department of Payment Certification of National Payment Certification Agency, Cambodia
4- Technical Department of National Pediatric Hospital, Cambodia

* umsamnang56@gmail.com

Abstract

Health insurance is essential in reducing or eliminating the financial constraint to accessing maternal health services caused by out-of-pocket payments. Also, it has a beneficial effect in minimizing the number of maternal and child mortality. However, limited studies in Cambodia examined the association between health insurance coverage on antenatal care (ANC) utilization. Therefore, this study examined the effect of health insurance coverage on ANC utilization in Cambodia. The study utilized data from the 2021-2022 Cambodia Demographic and Health Surveys (CDHS). A total of 3,162 weighted women who gave birth within two years were included in the study. Multiple logistic regression analysis was explored to assess the association between health insurance coverage on women who attended four or more ANC visits. About 24.9% of the women had health insurance coverage during 2021-2022. Most (86.1%) of women attended four or more ANC visits. Women with health insurance coverage were statistically significantly associated with attending four or more ANC visits with an adjusted odds ratio (AOR = 1.6; 95% CI: 1.1–2.4). Other covariates significantly associated with women who attended four or more ANC visits include women with higher education (AOR = 3.1; 95% CI: 1.2–7.7), secondary education (AOR = 2.3; 95% CI: 1.5–3.5), richest households (AOR = 3.2; 95% CI: 1.5-6.8), and richer households (AOR = 1.9;95% CI: 1.2-2.8). Pregnant women with health insurance coverage, who had completed at least secondary education and had a better wealth index were more likely to attend at least four ANC visits. Thus, improving health insurance coverage, and women's economic and education may be essential to improving women's access to attended ANC utilization services in Cambodia.

Key work: Health insurance, Antenatal care utilization, Pregnant women, Cambodia

Introduction

Cambodia's maternal mortality rate has significantly declined in the past decade. Data from the 2021-2022 Cambodia Demographic and Health Surveys (CDHS) show that maternal mortality had declined dramatically, from 488 to 154 per 100,000 live births between 2000 and 2021-2022 [1,2]. By 2030, the global maternal death ratio is expected to drop to less than 70 per 100,000 live births, according to Sustainable Development Goal (SDG) 3.1[3]. This achievement can be attributed to the country's concerted effort to increase women's access to maternal health services, particularly the initiative to increase institutional births [4]. Institutional births dramatically increased, from 19.3% to 98%, while the proportion of pregnant women attending four or more antenatal care (ANC) appointments increased considerably, from 9% to 86.1%, between 2000 and 2021-2022 [5]. In several studies, women who had health insurance had higher rates of using maternal health treatments, such as timely ANC and attending four or more ANC visits [6-9].

In 2019, the total population of Cambodia was 15.55 million, with 17.8% living below the national poverty line [10]. Since 2016, Cambodia has been classified as a lower-middle-income country, with gross domestic product (GDP) per capita from 302 US dollars in 2000 to 1,625 US dollars in 2021 [11]. Also, current expenditures on health per capita significantly increased from 20 US dollars in 2000 to 116 US dollars in 2020 [12]. Globally, 50% of people cannot access essential health services, as the World Bank and World Health Organization (WHO) reported in 2017 [13].

Cambodian National Social Security Fund (NSSF) has provided health insurance coverage to formal sector workers [14]. And poor households are covered by the Health Equity Fund (HEF), the co-financing mechanism of the government and development partners [15]. By 2025, the government intends to expand the reach of the NSSF health insurance program to include the entire population [16]. Data from CDHS 2021-2022 indicated that 22% of women and 13% of men aged 15-49 years have any health insurance, respectively [5]. Health insurance coverage is expected to provide financial risk protection and reduce disparities in access by facilitating greater uptake of maternal health services [13]. To our knowledge, limited published peer-reviewed studies assess the association between health insurance coverage and access to maternal health services among women of reproductive age in Cambodia using updated data. One prior study on health insurance

coverage and its impact on maternal healthcare utilization in low- and middle-income countries utilized data from CDHS 2010 [8]. This study included all women and men aged 15-49 and pooled Demographic and Health Survey (DHS) data in 30 low-and middle-income countries (LMICs) [8]. An additional study aimed to assess levels of health insurance coverage in 30 LMICs and examines the impact of health insurance status on the use of maternal health care in eight countries spanning sub-Saharan Africa (Burundi et al., Namibia, and Rwanda), West Asia (Albania), and South and Southeast Asia (Cambodia and Indonesia) [8]. Several pieces of evidence on the effect of health insurance coverage on ANC utilization have been published [6-9,17-19]; results indicate that women with health insurance coverage had higher odds attended four or more ANC visits than those without [6-9,17-19]. Moreover, those women who reported exposure to media, married women, those with high education, those living in wealthy economic families, those who are unemployed, and those living in urban areas were also factors associated with attending four or more ANC visits [6-9,17-19]. Given the lack of scholarship addressing this health concern among Cambodian women aged 15-49, we examined the effects of health insurance coverage on antenatal care (ANC) utilization among women who had a live birth in the past two years in Cambodia. The findings will provide a broader perspective on levels of health insurance coverage and the impact of health insurance status on the use of maternal health care in Cambodia. Additionally, the study will enable policymakers to understand better health insurance coverage among the adult population in Cambodia and proffer suggestions for improving universal health coverage in Cambodia.

Material and Methods

Ethical statement

The CDHS 2021–2022 is publicly available, with all personal identifiers of study participants removed. Permission to analyze the data was granted by registering with the DHS program website at (URL: https://dhsprogram.com/data/available-datasets.cfm). Written informed consent was obtained from the parent/guardian of each participant under 18 before data collection. The Cambodia National Ethics Committee for Human Health Research (NECHR) approved the data collection tools and procedures for CHDS 2021-2022 for Health Research on 10 May 2021

(Reference number: 83 NECHR) and ICF's Institutional Review Board (IRB) in Rockville, Maryland, USA.

Data source

We used data from the most recent CDHS (2021–2022), a household survey conducted every five years nationally representative of the population [5]. The two-stage stratified cluster sampling method collected the samples from all provinces. At the first stage, clusters, or enumeration areas (EAs), that represent the entire country (urban and rural), are randomly selected from the sampling frame using probability proportional (PPS) to cluster size. In the second stage, a complete listing of households was selected from each cluster using an equal probability systematic sampling, and then interviews were conducted with women aged 15–49 years who were born in the five years preceding the survey in the complete list selected households [5]. In total, 19,496 women aged 15-49 who had given birth in the last five years were interviewed face-to-face, using the survey standard questionnaire to collect information from women on several health indicators such as maternal health care service utilization, maternal and child health, nutrition, and reproductive health services [5]. Overall, 15,046 women who had not given birth in the past two years were excluded. Data restriction resulted in women who had a live birth in the past two years in a final analytic sample of 3,292 women (3,162 weighted women).

Measurements

Outcome variable

This study's outcome was the number of ANC visits during the last pregnancy among women aged 15-49 years (coded as 0 = less than 4 ANC visits included women who reported no ANC visits and 1 = four or more ANC visits) [6,18,20].

Independent variables

The primary independent variable is maternal health insurance coverage (coded as 0=no (reference and 1=yes), including public and private insurance. The confounding variables included maternal factors: Women’s age in years (coded as 1=15-30 (reference) and 2=31-49), marital status (coded as 1=married (reference) and 2=not married), birth order (coded as 1=1 (reference), 2=2-3, and

3=4 or more), education (coded as 0 = no education (reference), 1=primary, and 2=secondary or higher), occupation (coded as 0=not working (reference), 1=professional, 2=sales or services, 3=agricultural, and 4=manual labor). Individual Household factors included the household wealth index (coded as 1=poorest (reference), 2=poorest, 3=medium, 4=richer, and 5=richest) were calculated following the principal component analysis (PCA) [5]. Cambodia's geographical regions were grouped into four categories (coded as 1=Plains (reference), 2=Tonle Sap, 3=Coastal/Sea, and 4=Mountains), and place of residences (coded as 1=urban (reference) and 2=rural) was defined based on Cambodia's General Population Census 2019 and adapted from the original CDHS 2021-2022 [5,10].

Statistical analysis

Statistical analysis was performed using STATA version 17 (StataCorp LLC). We applied for the DHS standard sampling weight variable (**v005/1,000,000**). Then, we used the survey-specific STATA command "**svy**" for descriptive and analytical analysis. Women's socio-economic and demographic characteristics were described using weighted frequency and percentage distributions.

Bivariate analysis using Chi-square tests assessed the association between the variables of interest (maternal and individual household characteristics) and ANC visits. All independent variables associated with ANC use at p-value ≤ 0.10, or that had a potential confounder variable [6,18] were included in the multiple logistic regression analysis to determine the independent factors related to ANC use [26]. Multicollinearity between original independent variables was checked, including women's age, number of children ever born, education, wealth index, occupation, marital status, coverage health insurance, and place of residence. The result of the evaluating variance inflation factor (VIF) scores after fitting an Ordinary Least Squares regression model with the mean value of VIF was 1.53, which is less than the cutoff point indicating no collinearity correlation among the independent variables [27].

Results

Characteristics of the study population

Table 1 describes the socio-economic and demographic characteristics of the 3,162 women aged 15–49 born in the last two years preceding the survey. The mean age was 22.2 years old (SD = 4.2 years); the age group of 15–29 years old accounted for 94.3%. The majority (95%) were currently married. More than 33.4% of women had their first child. Half of the women completed at least secondary education, while 10.6% had no formal education. Only 6.5% of workers were professionals, and 31.2% were unemployed. Of the sample, 20.7% of women were from the poorest households, and 19.7% were from poorer households. Sixty-two percent of the women lived in rural areas. Only 786 (24.9%) women aged 15–49 had health insurance coverage. 86.1% of women attended at least four ANC visits during pregnancy.

Table 1. Socio-economic and demographic characteristics of women aged 15-49 years with a birth in the last two years preceding the survey (N = 3,162 weighted)

Variables	Freq.	%
**Mean age at the time of birth (SD)**	22.2(4.2)	
15-29	2,982	94.3
30-49	180	5.7
**Marital status**		
Married	3,004	95.0
Not married	158	5.0
**Birth order**		
1child	1055	33.4
2-3 children	1197	37.9
4 or more children	909	28.7
**Educational**		
No education	334	10.6
Primary	1253	39.6
Secondary	1361	43.0
Higher	214	6.8
**Occupation**		
Not working	986	31.2
Professional	205	6.5
Sales	579	18.3
Agricultural	445	14.1
Services	76	2.4
Manual labor	810	25.6
**Wealth index**		
Poorest	655	20.7

Poorer	623	19.7
Middle	626	19.8
Richer	683	21.6
Richest	574	18.2
**Residence**		
Urban	1202	38.0
Rural	1960	62.0
**Region**		
Plain	1532	48.5
Tonle Sap	996	31.5
Coastal	201	6.4
Plateau/Mountain	432	13.7
**Covered by health insurance**		
No	2376	75.1
Yes	786	24.9
**Number of ANC visits**		
< 4 ANC	440	13.9
≥ 4 ANC	2722	**86.1**

Notes: *Survey weights are applied to obtain weighted percentages. ****Plains:*** *Phnom Penh, Kampong Cham, Tbong Khmum, Kandal, Prey Veng, Svay Rieng, and Takeo;* ***Tonle Sap:*** *Banteay Meanchey, Kampong Chhnang, Kampong Thom, Pursat, Siem Reap, Battambang, Pailin, and Otdar Meanchey;* ***Coastal/sea:*** *Kampot, Kep, Preah Sihanouk, and Koh Kong;* ***Mountains:*** *Kampong Speu, Kratie, Preah Vihear, Stung Treng, Mondul Kiri, and Ratanak Kiri.*

Factors Associated with Four or more ANC visits in Chi-Square analysis

In bivariate analysis (**Table 3**), a higher proportion of women with health insurance coverage had a significant association with four or more ANC visits (91.6% vs. 84.2%, p < 0.001). Women aged 31–49 reported being more likely to attend four or more ANC visits (88.0% vs. 86.0%, p < 0.001). Also, married women reported four or more ANC visits than nonmarried women (86.6% vs. 76.2%, p = 0.007). Women with no education were less likely to attend four or more ANC visits than those with higher education (71.0% vs. 95.6%, p < 0.001). Four or more ANC visits were higher among women working in professional (95.3%) and service (96.4%), respectively, compared to unemployed women (84.3), with p <0.001). Additionally, four or more ANC visits were higher among women from the richer and richest on the wealth index (94.8 and 89.4%, respectively), compared to the poorer and poorest (73.9 and 86.9%, respectively, with p < 0.001). Lastly, women living in urban areas reported higher four or more ANC visits than in rural areas (91.5 vs. 82.7%, p < 0.001).

Table 3. Maternal and household characteristics by women attending at least four antenatal care and delivery in a health facility **(N = 3,162)**

Variables	Number of ANC visits		p-value
	**Four or more**	**Less than four**	
	**n=2,722**	**n=440**	
	%	%	
**Covered by health insurance**			
No	84.2	15.8	<0.001
Yes	91.6	8.4	
**Age at time of birth**			
15-30	86.0	14.0	<0.001
31-49	88.0	12.0	
**Marital status**			
Married	86.6	13.4	0.007
Not married	76.2	23.8	
**Birth order**			
1 child	88.4	11.6	<0.001
2-3 children	88.6	11.4	
4 or more children	80.1	19.9	
**Educational**			
No education	71.0	29.0	<0.001
Primary	84.1	15.9	
Secondary	90.1	9.9	
Higher	95.6	4.4	
**Occupation**			
Not working	84.3	15.7	<0.001
Professional	95.3	4.7	
Sales	84.4	15.6	
Agricultural	79.0	21.0	
Services	96.4	3.6	
Manual labor	89.9	10.1	
**Wealth index**			
Poorest	73.9	26.1	<0.001
Poorer	86.9	13.1	
Middle	86.3	13.7	
Richer	89.4	10.6	
Richest	94.8	5.2	
**Residence**			
Urban	91.5	8.5	<0.001
Rural	82.7	17.3	
**Region**			
Plain	89.3	10.7	<0.001
Tonle Sap	86.6	13.4	
Coastal	89.0	11.0	
Plateau/Mountain	72.2	27.8	

***Notes:** Survey weights are applied to obtain weighted percentages. ****Plains:** Phnom Penh, Kampong Cham, Tbong Khmum, Kandal, Prey Veng, Svay Rieng, and Takeo; **Tonle Sap:** Banteay Meanchey, Kampong Chhnang, Kampong Thom, Pursat, Siem Reap, Battambang, Pailin, and Otdar Meanchey; **Coastal/sea:** Kampot, Kep, Preah Sihanouk, and Koh Kong; **Mountains:** Kampong Speu, Kratie, Preah Vihear, Stung Treng, Mondul Kiri, and Ratanak Kiri.*

Association between health insurance and maternal Healthcare Services Utilization

Table 4 shows the results of the multiple logistic regression analysis of the association between health insurance coverage and maternal healthcare services utilization after controlling for the socio-demographic factors. Compared to women without health insurance, those with health insurance coverage were more likely to attend four or more ANC visits (AOR = 1.6, 95% CI: 1.1–2.4). Women with higher education (AOR = 3.1, 95% CI: 1.2–7.7), secondary education (AOR = 2.3, 95% CI: 1.5–3.5), and primary education (AOR = 1.7, 95% CI: 1.2–2.7) were more likely to have four or more ANC visits than women without any formal education. The odds of having four or more ANC visits were more significant for women from the wealthiest households than for those from the poorest households: richest households (AOR = 3.2; 95% CI: 1.5-6.8), richer households (AOR = 1.9; 95% CI: 1.2-2.8), and middle households (AOR = 1.5; 95% CI: 1.1-2.2). However, the odds of having four or more ANC visits were lower in unmarried women than in married women (AOR = 0.5; 95% CI: 0.3-0.8).

Table 4. Association between health insurance and four or more ANC visits in simple and multiple logistic regression model **(N = 3,162)**

Variables	Four or more ANC visits			
	Unadjusted		Adjusted	
	OR	95% CI	AOR	95% CI
**Covered by health insurance**				
No	Ref.		Ref.	
Yes	2.0***	(1.4-2.9)	**1.6***	**(1.1-2.4)**
**Age at time of birth**				
15-30	Ref.		Ref.	
31-49	1.2	(0.7-2.1)	1.1	(0.6-2.0)
**Marital status**				
Married	Ref.		Ref.	
Not married	0.5**	(0.3-0.8)	**0.5****	**(0.3-0.8)**
**Birth order**				
1child	Ref.		Ref.	
2-3 children	1.0	(0.8-1.4)	1.1	(0.8-1.5)
4 or more children	0.5***	(0.4-0.7)	0.7*	(0.5-1.0)
**Educational**				
No education	Ref.		Ref.	
Primary	2.2***	(1.5-3.1)	**1.8****	**(1.2-2.7)**
Secondary	3.7***	(2.6-5.3)	**2.3*****	**(1.5-3.5)**
Higher	8.9***	(4.3-18.3)	**3.1***	**(1.2-7.7)**
**Occupation**				
Not working	Ref.		Ref.	
Professional	3.8***	(2.0-7.0)	1.4	(0.7-2.8)

Sales	1.0	(0.7-1.4)	0.7	(0.5-1.0)
Agricultural	0.7*	(0.5-1.0)	1.0	(0.7-1.4)
Services	4.9**	(1.8-13.5)	2.6	(0.9-7.2)
Manual labor	1.7**	(1.2-2.3)	1.3	(0.9-1.9)
**Wealth index**				
Poorest	Ref.		Ref.	
Poorer	2.4***	(1.7-3.2)	**1.7****	**(1.2-2.3)**
Middle	2.2***	(1.6-3.1)	**1.5***	**(1.1-2.2)**
Richer	3.0***	(2.1-4.2)	**1.9****	**(1.2-2.8)**
Richest	6.4***	(3.4-12.0)	**3.2****	**(1.5-6.8)**
**Residence**				
Urban	Ref.		Ref.	
Rural	0.4***	(0.3-0.6)	0.8	(0.5-1.1)
**Region**				
Plain	Ref.		Ref.	
Tonle Sap	0.8	(0.6-1.1)	1.2	(0.9-1.7)
Coastal	1.0	(0.6-1.5)	1.3	(0.8-2.1)
Plateau/Mountain	0.3***	(0.2-0.4)	0.5***	(0.3-0.6)

Ref = reference value

* $p < 0.05$, ** $p < 0.01$, *** $p < 0.001$

Notes: *Survey weights are applied to obtain weighted percentages. ****Plains:*** *Phnom Penh, Kampong Cham, Tbong Khmum, Kandal, Prey Veng, Svay Rieng, and Takeo;* ***Tonle Sap:*** *Banteay Meanchey, Kampong Chhnang, Kampong Thom, Pursat, Siem Reap, Battambang, Pailin, and Otdar Meanchey;* ***Coastal/sea:*** *Kampot, Kep, Preah Sihanouk, and Koh Kong;* ***Mountains:*** *Kampong Speu, Kratie, Preah Vihear, Stung Treng, Mondul Kiri, and Ratanak Kiri.*

Discussion

We analyzed the most recent 2021–2022 CDHS data to examine the relationship between health insurance coverage and receiving four or more ANC visits during pregnancy. Overall, 24.9% of women reported having health insurance coverage among women of reproductive age who gave birth within two years of the survey. This finding is slightly similar to lower-middle-income countries, where 27.3% of women had health insurance coverage [19]. This is higher than in low-income countries, where 7.9% of women have health insurance coverage [19]. However, lower than in upper-middle-income countries, 52.5% of women had health insurance coverage [19]. Since the formal launch of the Cambodia National Social Security Fund (NSSF) with the Health Insurance Scheme in 2008, the proportion of women with health insurance coverage has increased from 16% in 2014 to 22% in 2021–2022 [5]. This proportion exponentially increased due to the Royal Government of Cambodia's implementation of the NSSF for all workers in the formal and informal sectors of the economy [10]. Moreover, it has plans to extend the healthcare benefits under the NSSF to the family members of the employees as well [16].

This study found that women with health insurance coverage were 1.6 times more likely to attend four or more ANC visits during pregnancy. Previous studies documented the positive relationship between health insurance and the number of ANC visits among women of reproductive age [8,9,19]. Health insurance eliminates the financial barrier to accessing maternal health services caused by out-of-pocket payments. It has a beneficial effect in reducing the number of low-birth-weight babies born and child mortality [17,21]. The result is more equitable access to care, potentially improving maternal health outcomes [9,19]. The MoH has since raised the minimum standard for ANC visits during pregnancy to at least four trips [20,22]. The dramatically significant increase in the highest prevalence of four or more ANC visits was an effort by the Royal Government of Cambodia, which has strengthened health facilities across the country, particularly in rural areas, improved infrastructure, provided essential medical equipment and supplies, increased the number of midwives, expanded antenatal care, and provided more skilled medical practitioners at childbirth to ensure safe delivery practices. Furthermore, to encourage early and routine ANC visits, the government is offering pregnant women a monetary incentive of 20 US dollars for each visit during a maximum of four ANC visits at any health facility with a contract with the National Social Security Fund (NSSF) [14,23].

This study found that increased education and household wealth index increased the likelihood of four or more ANC visits. Women's education and higher wealth index are more likely to attend four or more ANC visits. This aligns with previous evidence around socio-economic inequalities in maternal health service utilization in Cambodia and South Asia [24,25]. This may be because the more educated women is the more aware of the importance of ANC for their health and baby [26]. Additionally, education gives women the power to decide whether to seek medical attention and enables them to recognize warning signs of pregnancy complications. Moreover, women from higher-income households were more likely to be able to cover the costs of care-seeking, including any related expenses and transportation [24,25]. Thus, in this study, women with higher education levels and household wealth indexes had the highest proportion of health insurance coverage.

This study has several strengths. First, it used the most recent women's data from the 2021–2022 CDHS, an extensive representative national population-based household survey with a high response rate of 97%. Second, the recall bias has been minimized by limited analysis of women's

most recent deliveries within the last two years preceding the survey [5]. Third, the complex survey design and sampling weights in the analysis accounted for both descriptive and analytics methods that enabled us to generalize our findings to the population of WRA in Cambodia. In addition, DHS data were collected using validated survey methods and highly trained data collectors, contributing to improved data quality [27]. Last, to our knowledge, this is the first study to report the association between health insurance coverage and ANC visits in Cambodia. After controlling sociodemographic factors, we found significant associations between health insurance coverage and several forums or more ANC visits. A key finding compares the association with other studies in Southeast Asia and globally.

Despite this, there are several limitations. First, this study used a secondary analysis, so it did not address health institution factors of antenatal care utilization and service availability; hence, this study could not explore the quality of ANC services, though the quality of healthcare services plays a vital role in patient satisfaction and use. Second, the study's cross-sectional nature could not assist in the temporal relationship of variables including the number of years since women joined health insurance on ANC utilization. Therefore, further study should be conducted to identify factors related to health institutions. In addition, antenatal care utilization should be performed based on the new WHO guidelines revised in 2016 at the national level [22]. Moreover, longitudinal studies that address comprehensive variables should be studied. Third, we excluded other factors, such as maternal complications and women's empowerment indicators, that could affect the use of maternal care. Lastly, CDHS did not assess a direct measure of maternal health literacy.

Conclusion

This is the first study to report the association between health insurance coverage and ANC visits in the healthcare setting in Cambodia. Cambodian pregnant women attend four or more antenatal care visits, which is slightly high. However, it still needs to be satisfactory. Health among coverage women of reproductive age in Cambodia is relatively low. Moreover, we found that women with health insurance, women with education, and being rich in the wealth quintile were strong predictors of women attending four or more ANC visits. There is a need to pay close attention to improving the uptake of health insurance among women of reproductive age, especially targeting women with no education, from low-income families, and women who reside in rural areas. Policymakers may need to prioritize women of reproductive age in designing and implementing health insurance programs to increase their uptake. This would provide financial risk protection, facilitate access to maternal health services, and possible attainment of Cambodia’s SDG 3 targets.

Acknowledgments

The authors would like to thank DHS-ICF, who approved the data used for this paper.

References

1. National Institute of Statistics, Directorate General for Health, and ICF International. Cambodia Demographic and Health Survey 2014. Phnom Penh, Cambodia, and Rockville, Maryland, USA: National Institute of Statistics, Directorate General for Health, and ICF International. Available online: https://dhsprogram.com/pubs/pdf/fr312/fr312.pdf. 2015.
2. National Institute of Statistics PP, Cambodia, Ministry of Health Phnom Penh, Cambodia, The DHS Program, ICF, Rockville, Maryland, USA. Cambodia Demographic and Health Survey 2021–22. Phnom Penh, Cambodia, and Rockville, Maryland, USA: NIS, MoH, and ICF. 2022. Available from: https://dhsprogram.com/pubs/pdf/FR377/FR377.pdf.
3. The United Nations Development Programme. The Sustainable Development Goals by 2016-2030. Available from: https://www.undp.org/content/undp/en/home/sustainable-development-goals.html.
4. Acuin CS, Khor GL, Liabsuetrakul T, Achadi EL, Htay TT, Firestone R, et al. Maternal, neonatal, and child health in southeast Asia: towards greater regional collaboration. Lancet. 2011;377(9764):516-25. Epub 20110125. doi: 10.1016/s0140-6736(10)62049-1. PubMed PMID: 21269675; PubMed Central PMCID: PMCPMC7159081.
5. National Institute of Statistics (NIS) MoHM, and ICF. . Cambodia Demographic and Health Survey 2021–22 Final Report. Phnom Penh, Cambodia, and Rockville, Maryland, USA: NIS, MoH, and ICF. 2023. Available from: https://dhsprogram.com/pubs/pdf/PR136/PR136.pdf.
6. Merga BT, Raru TB, Deressa A, Regassa LD, Gamachu M, Negash B, et al. The effect of health insurance coverage on antenatal care utilizations in Ethiopia: evidence from national survey. Front Health Serv. 2023;3:1101164. Epub 20231006. doi: 10.3389/frhs.2023.1101164. PubMed PMID: 37869683; PubMed Central PMCID: PMCPMC10587574.
7. Aboagye RG, Okyere J, Ahinkorah BO, Seidu AA, Zegeye B, Amu H, et al. Health insurance coverage and timely antenatal care attendance in sub-Saharan Africa. BMC Health Serv Res. 2022;22(1):181. Epub 20220211. doi: 10.1186/s12913-022-07601-6. PubMed PMID: 35148769; PubMed Central PMCID: PMCPMC8840787.
8. Wang W, Gheda Temsah, and Lindsay Mallick. . Health Insurance Coverage and Its Impact on Maternal Health Care Utilization in Low- and Middle-Income Countries. DHS Analytical Studies No. 45. Rockville, Maryland, USA: ICF International. . 2014.
9. Ahuru RR, Omon IJ, Nzoputam CI, Ekomoezor E. Health insurance ownership and maternal health service uptake among Nigerian women. HEALTH. 2021;12.
10. National Institute of Statistics. Ministry of Planning. General population census of the Kingdom of Cambodia 2019. Available from: https://www.nis.gov.kh/nis/Census2019/Final%20General%20Population%20Census%202019-English.pdf.
11. The World Bank In Cambodia. Overview 2019. Available from: https://www.worldbank.org/en/country/cambodia/overview.
12. World Health Organization (WHO). Global Health Expenditure Database 2019. Available from: https://apps.who.int/nha/database/PHC_Country_profile/Index/en.
13. World Health Organization (WHO); World Bank and WHO. Half the world lacks access to essential health services, 100 million still pushed into extreme poverty because of health expenses 2017. 2017. Available from: http://www.who.int/mediacentre/news/releases/2017/half-lacks-access/en/.
14. National Social Security Fund (NSSF). Health Insurance Scheme 2016. Available from: https://www.nssf.gov.kh/health-care-scheme-2/.
15. Nakamura H, Amimo F, Yi S, Tuot S, Yoshida T, Tobe M, et al. Implementing a sustainable health insurance system in Cambodia: a study protocol for developing and validating an efficient household income-level assessment model for equitable premium collection. Int J Equity Health. 2020;19(1):17. Epub 20200131. doi: 10.1186/s12939-020-1126-8. PubMed PMID: 32005237; PubMed Central PMCID: PMCPMC6995079.
16. The Royal Government of Cambodia. National Social Protection Policy Framework 2016–2025. Phnom Penh: The Council of Ministers; 2017.

17. Likka MH, Handalo DM, Weldsilase YA, Sinkie SO. The effect of community-based health insurance schemes on utilization of healthcare services in low- and middle-income countries: a systematic review protocol of quantitative evidence. JBI Database System Rev Implement Rep. 2018;16(3):653-61. doi: 10.11124/jbisrir-2017-003381. PubMed PMID: 29521866.
18. Dadjo J, Ahinkorah BO, Yaya S. Health insurance coverage and antenatal care services utilization in West Africa. BMC Health Serv Res. 2022;22(1):311. Epub 20220307. doi: 10.1186/s12913-022-07698-9. PubMed PMID: 35255895; PubMed Central PMCID: PMCPMC8899447.
19. Hooley B, Afriyie DO, Fink G, Tediosi F. Health insurance coverage in low-income and middle-income countries: progress made to date and related changes in private and public health expenditure. BMJ Glob Health. 2022;7(5). doi: 10.1136/bmjgh-2022-008722. PubMed PMID: 35537761; PubMed Central PMCID: PMCPMC9092126.
20. Ministry of Health. Guideline for implementation the service package of Antenatal Care Delivery and Post natal Care 2019 [updated 2019]. 3:[Available from: https://niph.org.kh/niph/uploads/library/pdf/GL220_NMCHC_anc_pnc_package_eng.pdf.
21. Imo CK, De Wet-Billings N, Isiugo-Abanihe UC. The impact of maternal health insurance coverage and adequate healthcare services utilisation on the risk of under-five mortality in Nigeria: a cross-sectional study. Arch Public Health. 2022;80(1):206. Epub 20220913. doi: 10.1186/s13690-022-00968-2. PubMed PMID: 36100949; PubMed Central PMCID: PMCPMC9472384.
22. WHO Guidelines Approved by the Guidelines Review Committee. WHO Recommendations on Antenatal Care for a Positive Pregnancy Experience. Geneva: World Health Organization

Copyright © World Health Organization 2016.; 2016.
23. Ministry of Information. Government Raises Allowances For Mothers And Children. Available from: https://www.information.gov.kh/articles/108509.
24. Chham S, Radovich E, Buffel V, Ir P, Wouters E. Determinants of the continuum of maternal health care in Cambodia: an analysis of the Cambodia demographic health survey 2014. BMC Pregnancy Childbirth. 2021;21(1):410. Epub 20210602. doi: 10.1186/s12884-021-03890-7. PubMed PMID: 34078318; PubMed Central PMCID: PMCPMC8170811.
25. Lee HY, Oh J, Kim R, Subramanian SV. Long-term trend in socioeconomic inequalities and geographic variation in the utilization of antenatal care service in India between 1998 and 2015. Health Serv Res. 2020;55(3):419-31. Epub 20200304. doi: 10.1111/1475-6773.13277. PubMed PMID: 32133652; PubMed Central PMCID: PMCPMC7240766.
26. Thakkar N, Alam P, Saxena D. Factors associated with underutilization of antenatal care in India: Results from 2019-2021 National Family Health Survey. PLoS One. 2023;18(5):e0285454. Epub 20230508. doi: 10.1371/journal.pone.0285454. PubMed PMID: 37155665; PubMed Central PMCID: PMCPMC10166529.
27. Allen C, Fleuret J, Ahmed J, editors. Data quality in demographic and health surveys that used long and short questionnaires2020: ICF.

Click here to access/download
Supporting Information
PLOSOne_Human_Subjects_Research_Checklist.docx

The effect of h~~H~~ealth insurance coverage ~~and~~ on antenatal care utilization in Cambodia: A secondary analysis of Cambodia Demographic and Health Survey 2021-2022

Samnang Um[1,2*], Channnarong Phan[1], Khun Veha[3], Soklim Pay[4], Darapheak Chau[1]

1- National Institute of Public Health, Cambodia
2- Faculty of Social Science and Humanities, Royal University of Phnom Penh, Cambodia
3- Department of Payment Certification of National Payment Certification Agency, Cambodia
4- Technical Department of National Pediatric Hospital, Cambodia

* umsamnang56@gmail.com

Abstract

Health insurance is essential in reducing or eliminating the financial constraint to accessing maternal health services caused by out-of-pocket payments. Also, i~~I~~t has a beneficial effect in minimizing the number of maternal and child mortality. However, limited studies in Cambodia examined the association between health insurance coverage on antenatal care (ANC) utilization. Therefore, this study was~~We~~ examined the ~~impact~~ effect of health insurance coverage ~~and~~ on ~~antenatal care (~~ANC~~)~~ utilization in Cambodia. The study utilized ~~We used~~ data from the 2021-2022 Cambodia Demographic and Health Surveys (CDHS). A total of 3,162 weighted women who gave birth within two years were included in the study. Multiple logistic regression analysis was explored to assess t~~T~~he association between health insurance coverage ~~and~~ on ~~with pregnant~~ women who attended ~~at least~~ four or more ~~antenatal care~~ANC visits. ~~during their pregnancy was evaluated using multiple logistic regression analysis~~. About 24.9% of the women had health insurance coverage during 2021-2022. Most (86.1%) of women attended ~~at least~~ four~~4~~ or more ANC visits. ~~Almost 91.6% of women participating in four or more ANC visits were covered by health insurance.~~ Women with ~~Having~~ health insurance coverage ~~and attending four or more ANC visits~~ were statistically significantly associated with ~~women~~ attending ~~at least~~ four or more ANC visits with an adjusted odds ratio (AOR = 1.6; 95% CI: 1.1–2.4). Other covariates ~~factors~~ significantly associated with women attended ~~ance of~~ ~~at~~ four or more ANC visits include women with higher education (AOR = 3.1; 95% CI: 1.2–7.7), secondary education (AOR = 2.3; 95% CI: 1.5–3.5), richest households (AOR = 3.2; 95% CI: 1.5-6.8), and richer households (AOR = 1.9;95% CI: 1.2-2.8). Pregnant women with health insurance coverage, who had completed at least secondary education and had a better wealth index were more likely to attend at least four ANC

visits. Thus, improving providing health insurance coverage, women's economic and education may be essential to improving women's access to maternal healthattended ANC utilization services in Cambodia.

Key work: Health insurance, Antenatal care utilization, Pregnant women, Cambodia

Introduction

Cambodia's maternal mortality rate has significantly declined in the past decade. Data from the 2021-2022 Cambodia Demographic and Health Surveys (CDHS) show that maternal mortality had declined dramatically, from 488 to 154 per 100,000 live births between 2000 and 2021-2022 [1,2]. By 2030, the global maternal death ratio is expected to drop to less than 70 per 100,000 live births, according to Sustainable Development Goal (SDG) 3.1 [3]. This achievement can be attributed to the country's concerted effort to increase women's access to maternal health services, particularly the initiative to increase institutional births [4]. Institutional births dramatically increased, from 19.3% to 98%, while the proportion of pregnant women attending four or more antenatal care (ANC) appointments increased considerably, from 9% to 86.1%, between 2000 and 2021-2022 [5]. In several studies, women who had health insurance had higher rates of using maternal health treatments, such as timely ANC and attending four or more ANC visits [6-9].

In 2019, the total population of Cambodia was 15.55 million, with 17.8% living below the national poverty line [10]. Since 2016, Cambodia has been classified as a lower-middle-income country, with gross domestic product (GDP) per capita from 302 US dollars in 2000 to 1,625 US dollars in 2021 [11]. Also, current expenditures on health per capita significantly increased from 20 US dollars in 2000 to 116 US dollars in 2020 [12]. Globally, 50% of people cannot access essential health services, as the World Bank and World Health Organization (WHO) reported in 2017 [13].

Cambodian National Social Security Fund (NSSF) has provided health insurance coverage to formal sector workers [14]. And poor households are covered by the Health Equity Fund (HEF), the co-financing mechanism of the government and development partners [15]. By 2025, the government intends to expand the reach of the NSSF health insurance program to include the entire

population [16]. Data from CDHS 2021-2022 indicated that 22% of women and 13% of men aged 15-49 years have any health insurance, respectively ~~Thus, nearly 70% of the population aged 15-49 reported not covered by health insurance~~ [5]. Health insurance coverage is expected to provide financial risk protection and reduce disparities in access by facilitating greater uptake of maternal health services [13]. To our knowledge, limited published peer-reviewed studies assess the association between health insurance coverage and access to maternal health services among women of reproductive age in Cambodia using updated data. One prior study on health insurance coverage and its impact on maternal healthcare utilization in low- and middle-income countries utilized data from CDHS 2010 [8]. This study included all women and men aged 15-49 and pooled Demographic and Health Survey (DHS) data in 30 low-and middle-income countries (LMICs) [8]. An additional study aimed to assess levels of health insurance coverage in 30 LMICs and examines the impact of health insurance status on the use of maternal health care in eight countries spanning sub-Saharan Africa (Burundi et al., Namibia, and Rwanda), West Asia (Albania), and South and Southeast Asia (Cambodia and Indonesia) [8]. Several pieces of evidence on the effect of health insurance coverage on ANC utilization have been published [6-9,17-19]; results indicate that women with health insurance coverage had higher odds attended four or more ANC visits than those without [6-9,17-19]. Moreover, those women who reported exposure to media, married women, those with high education, those living in wealthy economic families, those who are unemployed, and those living in urban areas were also factors associated with attending four or more ANC visits [6-9,17-19]. Given the lack of scholarship addressing this health concern among Cambodian women aged 15-49, we examined the effects of health insurance coverage ~~and~~ on antenatal care (ANC) utilization among ~~pregnant~~ women who had a live birth in the past two years in Cambodia. The findings will provide a broader perspective on levels of health insurance coverage and the impact of health insurance status on the use of maternal health care in Cambodia. Additionally, the study will enable policymakers to understand better health insurance coverage among the adult population in Cambodia and proffer suggestions for improving universal health coverage in Cambodia.

Material and Methods

Ethical statement

The CDHS 2021–2022 is publicly available, with all personal identifiers of study participants removed. Permission to analyze the data was granted by registering with the DHS program website at (URL: https://dhsprogram.com/data/available-datasets.cfm). Written informed consent was obtained from the parent/guardian of each participant under 18 before data collection. The Cambodia National Ethics Committee for Human Health Research (NECHR) approved the data collection tools and procedures for CHDS 2021-2022 for Health Research on 10 May 2021 (Reference number: # 83 NECHR) and ICF's Institutional Review Board (IRB) in Rockville, Maryland, USA.

Data source

We used data from the most recent CDHS (2021–2022), a household survey conducted every five years nationally representative of the population [5]. The two-stage stratified cluster sampling method collected the samples from all provinces. At the first stage, clusters, or enumeration areas (EAs), that represent the entire country (urban and rural), are randomly selected from the sampling frame using probability proportional (PPS) to cluster size. In the second stage, a complete listing of households was selected from each cluster using an equal probability systematic sampling, and then interviews were conducted with women aged 15–49 years who were born in the five years preceding the survey in the complete list selected households [5]. In total, 19,496 women aged 15-49 who had given birth in the last five years were interviewed face-to-face, using the survey standard questionnaire to collect information from women on several health indicators such as maternal health care service utilization, maternal and child health, nutrition, and reproductive health services [5]. Overall, 15,046 women who had not given birth in the past two years were excluded. Data restriction resulted in women who had a live birth in the past two years in a final analytic sample of 3,292 women (3,162 weighted women).

Measurements

Outcome variable

This study's outcome was the number of ANC visits during the last pregnancy among women aged 15-4~~5~~9 years (coded as 0 = less than 4 ANC visits included women who reported no ANC visits and 1 = four or ~~and~~ more ANC visits) [6,18,20].

Independent variables

The primary independent variable is maternal health insurance coverage (coded as 0=no (reference and 1=yes ~~vs. no~~), including public and private insurance. The confounding variables included maternal factors: Women's age in years (coded as 1=15-30 (reference) and 2=31-49), m~~M~~arital status (coded as 1=married (reference) and 2=not married), b~~B~~irth order (coded as 1=1 (reference)~~st~~, 2=2 ~~nd,~~ 3 ~~rd~~, and 3=4th or more), e~~E~~ducation (coded as 0 = no education (reference), 1=primary, and 2=secondary or higher), o~~O~~ccupation (coded as 0=not working (reference), 1=professional, 2=sales~~,~~ or services, 3=agricultural, and 4=manual labor).~~,~~ Individual Household factors included the h~~H~~ousehold wealth index (coded as 1=poorest (reference), 2=poorest, 3=medium, 4=richer, and 5=richest) were calculated following the principal component analysis (PCA) [5]. Cambodia's g~~G~~eographical regions were grouped into four categories ~~-~~(coded as 1=Plains (reference), 2=Tonle Sap, 3=~~-~~Coastal/Sea, and 4=Mountains), and p~~P~~lace of r~~R~~esidences (coded as 1=urban (reference) and 2=rural) was defined ~~(rural vs. urban)~~ based on Cambodia's General Population Census 2019 and adapted from the original CDHS 2021-2022 [5,10].

Statistical analysis

~~Analyses~~ Statistical analysis ~~were~~ was performed using STATA V17 (StataCorp LLC). We applied for the ~~C~~DHS standard sampling weight variable (***v005/1,000,000***). Then, we used the survey-specific STATA command "***svy***" for descriptive and analytical analysis. Women's socio-economic and demographic characteristics were described using weighted frequency and percentage distributions.

Bivariate analysis using Chi-square tests assessed the association between the variables of interest (maternal and individual household characteristics) and ANC use. All independent variables associated with ANC use at p-value ≤ 0.10, or that had a potential confounder variable [6,18] were included in the multiple logistic regression analysis to determine the independent factors related to ANC use [26]. Multicollinearity between original independent variables was checked, including

Formatted: Superscript

Formatted: Font: Bold, Italic

Formatted: Font: Bold, Italic

women's age, number of children ever born, education, wealth index, occupation, marital status, coverage health insurance, and place of residence. The r~~R~~esult of the evaluating variance inflation factor (VIF) scores after fitting an Ordinary Least Squares regression model with~~.~~ t~~T~~he mean value of VIF was 1.53, which is less than the cutoff point indicating no collinearity correlation among the independent variables [27].

Results

Characteristics of the study population

Table 1 describes the socio-economic and demographic characteristics of the 3,162 women aged 15–49 born in the last two years preceding the survey. The mean age was 22.2 years old (SD = 4.2 years); the age group of 15–29 years old accounted for 94.3%. The majority (95%) were currently married. More than 33.4% of women ~~were giving in~~had the first ~~birth order~~child. Half of the women completed at least secondary education, while 10.6% had no formal education. Only 6.5% of workers were~~are~~ professionals, and 31.2% were~~are~~ unemployed. Of the sample, 20.7% of women were from the poorest households, and 19.7% were from poorer households~~families~~. Sixty-two percent of the women lived in rural areas. Only 786 (24.9%) women aged 15–49 had health insurance coverage. 86.1% of women attended at least four ~~4~~ ANC visits during pregnancy.

Table 1. Socio-economic and demographic characteristics of women aged 15-49 years with a birth in the last two years preceding the survey (N = 3,162 weighted)

Variables	Freq.	%
**Mean age at the time of birth (SD)**	22.2(4.2)	
15-29	2,982	94.3
30-49	180	5.7
**Marital status**		
Married	3,004	95.0
Not married	158	5.0
**Birth order**		
1~~st~~ child	1055	33.4
2-3~~nd~~ children	1197	37.9
4~~3rd~~ ~~child~~ or more children	909	28.7
**Educational**		
No education	334	10.6
Primary	1253	39.6
Secondary	1361	43.0
Higher	214	6.8
**Occupation**		
Not working ~~None~~	986	31.2
Professional	205	6.5
Sales	579	18.3
Agricultural	445	14.1
Services	76	2.4
Manual labor	810	25.6
**Wealth index**		
Poorest	655	20.7
Poorer	623	19.7
Middle	626	19.8

Formatted: Not Superscript/ Subscript

Formatted: Font: 10 pt, Font color: Black, (Asian) Japanese, Ligatures: None

Richer	683	21.6
Richest	574	18.2
**Residence**		
Urban	1202	38.0
Rural	1960	62.0
**Region**		
Plain	1532	48.5
Tonle Sap	996	31.5
Coastal	201	6.4
Plateau/Mountain	432	13.7
**Covered by health insurance**		
No	2376	75.1
Yes	786	24.9
**Number of ANC visits**		
< 4 ANC	440	13.9
≥ 4 ANC	2722	**86.1**

Notes: *Survey weights are applied to obtain weighted percentages. ****Plains:*** *Phnom Penh, Kampong Cham, Tbong Khmum, Kandal, Prey Veng, Svay Rieng, and Takeo;* ***Tonle Sap:*** *Banteay Meanchey, Kampong Chhnang, Kampong Thom, Pursat, Siem Reap, Battambang, Pailin, and Otdar Meanchey;* ***Coastal/sea:*** *Kampot, Kep, Preah Sihanouk, and Koh Kong;* ***Mountains:*** *Kampong Speu, Kratie, Preah Vihear, Stung Treng, Mondul Kiri, and Ratanak Kiri.*

Formatted Table

~~Maternal health insurance characteristics~~

~~The highest proportion of women having health insurance was among those aged 15-30 years (25.3.4%, p = 0.094); higher education (48.1%, p < 0.001); worked in professional jobs (59.0%), manual labor (52.5%) with p < 0.001); and women from the richer households (30.2%), richest households (32.2%) with p <0.001); and women in urban areas (35.9%, p <0.001); **(see Table 2)**.~~

~~**Table 2.** Socio-economic and demographic characteristics by the health insurance coverage of women aged 15-49 years with a birth in the last two years preceding the survey (**N = 3,162**)~~

	Covered by health insurance				
Variables	No (N=2,367)		Yes (N=786)		p-value
**Age at time of birth**	**Freq.**	%	**Freq.**	%	
15-30	2,229	74.7	753	25.3	0.094
31-49	147	81.7	33	18.3	
**Marital status**					
Married	2,260	75.2	744	24.8	0.757
Not married	116	73.4	42	26.6	
**Birth order**					
1st child	778	73.7	277	26.3	0.035
2nd or 3rd child	876	73.2	321	26.8	
4th child or more	722	79.4	188	20.7	
**Educational**					
No education	269	80.5	65	19.5	<0.001

Primary	950	75.8	304	24.3	
Secondary	1,046	76.9	315	23.1	
Higher	111	51.9	103	48.1	
**Occupation**					
None	892	90.5	93	9.4	<0.001
Professional	84	41.0	121	59.0	
Sales	514	88.8	65	11.2	
Agricultural	387	87.0	58	13.0	
Services	64	84.2	12	15.8	
Manual labor	385	47.5	425	52.5	
**Wealth index**					
Poorest	537	82.0	118	18.0	<0.001
Poorer	493	79.1	131	21.0	
Middle	479	76.5	147	23.5	
Richer	477	69.8	206	30.2	
Richest	389	67.8	185	32.2	
**Residence**					
Urban	770	64.1	432	35.9	<0.001
Rural	1,606	81.9	354	18.1	
**Region**					
Plain	1,119	73.0	413	27.0	0.019
Tonle Sap	781	78.4	216	21.7	
Coastal	167	83.1	34	16.9	
- Plateau/Mountain	309.0	71.5	123.0	28.5	-

Notes: *Survey weights are applied to obtain weighted percentages. ****Plains:*** *Phnom Penh, Kampong Cham, Tbong Khmum, Kandal, Prey Veng, Svay Rieng, and Takeo;* ***Tonle Sap:*** *Banteay Meanchey, Kampong Chhnang, Kampong Thom, Pursat, Siem Reap, Battambang, Pailin, and Otdar Meanchey;* ***Coastal/sea:*** *Kampot, Kep, Preah Sihanouk, and Koh Kong;* ***Mountains:*** *Kampong Speu, Kratie, Preah Vihear, Stung Treng, Mondul Kiri, and Ratanak Kiri.*

Formatted Table

Factors Associated with Four or more ANC visits in Chi-Square analysis

In bivariate analysis (**Table 3**), a higher proportion of women with health insurance coverage had a significant association with four or more ANC visits (91.6% vs. 84.2%, $p < 0.001$). Women aged 31–49 reported being more likely to attend four or more ANC visits (88.0% vs. 86.0%, $p < 0.001$). Also, married women reported four or more ANC visits than nonmarried women (86.6% vs. 76.2%, $p = 0.007$). Women with no education were less likely to attend four or more ANC visits than those with higher education (71.0% vs. 95.6%, $p < 0.001$). Four or more ANC visits were higher among women working in professional (95.3%) and service (96.4%), respectively, compared to unemployed women (84.3), with $p < 0.001$). Additionally, four or more ANC visits were higher among women from the richer and richest on the wealth index (94.8 and 89.4%, respectively), compared to the poorer and poorest (73.9 and 86.9%, respectively, with $p < 0.001$). Lastly, women living in urban areas reported higher four or more ANC visits than in rural areas (91.5 vs. 82.7%, $p < 0.001$).

Table 3. Maternal and household characteristics by women attending at least four antenatal care and delivery in a health facility **(N = 3,162)**

**Variables**	**Number of ANC visits**		**p-value**
	**Four or more**	**Less than four**	
	**n=2,722**	**n=440**	
	%	%	
**Covered by health insurance**			
No	84.2	15.8	<0.001
Yes	91.6	8.4	
**Age at time of birth**			
15-30	86.0	14.0	<0.001
31-49	88.0	12.0	
**Marital status**			
Married	86.6	13.4	0.007
Not married	76.2	23.8	
**Birth order**			
1st child	88.4	11.6	<0.001
2~~-nd or~~ 3~~rd~~ children	88.6	11.4	
4~~th child~~ or more children	80.1	19.9	
**Educational**			
No education	71.0	29.0	<0.001
Primary	84.1	15.9	
Secondary	90.1	9.9	
Higher	95.6	4.4	
**Occupation**			
Not working~~ne~~	84.3	15.7	<0.001

Professional	95.3	4.7	
Sales	84.4	15.6	
Agricultural	79.0	21.0	
Services	96.4	3.6	
Manual labor	89.9	10.1	
**Wealth index**			
Poorest	73.9	26.1	<0.001
Poorer	86.9	13.1	
Middle	86.3	13.7	
Richer	89.4	10.6	
Richest	94.8	5.2	
**Residence**			
Urban	91.5	8.5	<0.001
Rural	82.7	17.3	
**Region**			
Plain	89.3	10.7	<0.001
Tonle Sap	86.6	13.4	
Coastal	89.0	11.0	
Plateau/Mountain	72.2	27.8	

***Notes:** Survey weights are applied to obtain weighted percentages. *__Plains:__ Phnom Penh, Kampong Cham, Tbong Khmum, Kandal, Prey Veng, Svay Rieng, and Takeo; __Tonle Sap:__ Banteay Meanchey, Kampong Chhnang, Kampong Thom, Pursat, Siem Reap, Battambang, Pailin, and Otdar Meanchey; __Coastal/sea:__ Kampot, Kep, Preah Sihanouk, and Koh Kong; __Mountains:__ Kampong Speu, Kratie, Preah Vihear, Stung Treng, Mondul Kiri, and Ratanak Kiri.*

Association between health insurance and maternal Healthcare Services Utilization

Table 4 shows the results of the multiple logistic regression analysis of the association between health insurance coverage and maternal healthcare services utilization after controlling for the socio-demographic factors. Compared to women without health insurance, those with health insurance coverage were more likely to attend four or more ANC visits (AOR = 1.6, 95% CI: 1.1–2.4). Women with higher education (AOR = 3.1, 95% CI: 1.2–7.7), secondary education (AOR = 2.3, 95% CI: 1.5–3.5), and primary education (AOR = 1.7, 95% CI: 1.2–2.7) were more likely to have four or more ANC visits than women without any formal education. The odds of having four or more ANC visits were more significant for women from the wealthiest households than for those from the poorest households: richest households (AOR = 3.2; 95% CI: 1.5-6.8), richer households (AOR = 1.9; 95% CI: 1.2-2.8), and middle households (AOR = 1.5; 95% CI: 1.1-2.2). However, the odds of having four or more ANC visits were lower in unmarried women than in married women (AOR = 0.5; 95% CI: 0.3-0.8).

Table 4. Association between health insurance and four or more ANC visits in simple and multiple logistic regression model **(N = 3,162)**

**Variables**	**Four or more ANC visits**			
	**Unadjusted**		**Adjusted**	
	**OR**	**95% CI**	**AOR**	**95% CI**
**Covered by health insurance**				
No	Ref.~~1.0~~		Ref.~~1.0~~	
Yes	2.0***	(1.4-2.9)	**1.6***	**(1.1-2.4)**
**Age at time of birth**				
15-30	Ref.~~1.0~~		Ref.~~1.0~~	
31-49	1.2	(0.7-2.1)	1.1	(0.6-2.0)
**Marital status**				
Married	Ref.~~1.0~~		Ref.~~1.0~~	
Not married	0.5**	(0.3-0.8)	**0.5****	**(0.3-0.8)**
**Birth order**				
1~~st~~ child	Ref.~~1.0~~		Ref.~~1.0~~	
2~~nd~~ ~~or~~ 3~~rd~~ children	1.0	(0.8-1.4)	1.1	(0.8-1.5)
4~~th~~ ~~child~~ or more children	0.5***	(0.4-0.7)	0.7*	(0.5-1.0)
**Educational**				
No education	Ref.~~1.0~~		Ref.~~1.0~~	
Primary	2.2***	(1.5-3.1)	**1.8****	**(1.2-2.7)**
Secondary	3.7***	(2.6-5.3)	**2.3*****	**(1.5-3.5)**
Higher	8.9***	(4.3-18.3)	**3.1***	**(1.2-7.7)**
**Occupation**				
Not working ~~None~~	Ref.~~1.0~~		Ref.~~1.0~~	
Professional	3.8***	(2.0-7.0)	1.4	(0.7-2.8)

Sales	1.0	(0.7-1.4)	0.7	(0.5-1.0)
Agricultural	0.7^{*}	(0.5-1.0)	1.0	(0.7-1.4)
Services	4.9^{**}	(1.8-13.5)	2.6	(0.9-7.2)
Manual labor	1.7^{**}	(1.2-2.3)	1.3	(0.9-1.9)
**Wealth index**				
Poorest	Ref.~~1.0~~		Ref.~~1.0~~	
Poorer	2.4^{***}	(1.7-3.2)	**1.7^{**}**	**(1.2-2.3)**
Middle	2.2^{***}	(1.6-3.1)	**1.5^{*}**	**(1.1-2.2)**
Richer	3.0^{***}	(2.1-4.2)	**1.9^{**}**	**(1.2-2.8)**
Richest	6.4^{***}	(3.4-12.0)	**3.2^{**}**	**(1.5-6.8)**
**Residence**				
Urban	Ref.~~1.0~~		Ref.~~1.0~~	
Rural	0.4^{***}	(0.3-0.6)	0.8	(0.5-1.1)
**Region**				
Plain	Ref.~~1.0~~		Ref.~~1.0~~	
Tonle Sap	0.8	(0.6-1.1)	1.2	(0.9-1.7)
Coastal	1.0	(0.6-1.5)	1.3	(0.8-2.1)
Plateau/Mountain	0.3^{***}	(0.2-0.4)	0.5^{***}	(0.3-0.6)

Ref = reference value

* $p < 0.05$, ** $p < 0.01$, *** $p < 0.001$

Notes: *Survey weights are applied to obtain weighted percentages. ****Plains:*** *Phnom Penh, Kampong Cham, Tbong Khmum, Kandal, Prey Veng, Svay Rieng, and Takeo;* ***Tonle Sap:*** *Banteay Meanchey, Kampong Chhnang, Kampong Thom, Pursat, Siem Reap, Battambang, Pailin, and Otdar Meanchey;* ***Coastal/sea:*** *Kampot, Kep, Preah Sihanouk, and Koh Kong;* ***Mountains:*** *Kampong Speu, Kratie, Preah Vihear, Stung Treng, Mondul Kiri, and Ratanak Kiri.*

Discussion

We analyzed the most recent 2021–2022 CDHS data to examine the relationship between health insurance coverage and receiving four or more ANC visits during pregnancy. Overall, 24.9% of women reported having health insurance coverage among women of reproductive age who gave birth within two years of the survey. This finding is slightly similar to lower-middle-income countries, where 27.3% of women had health insurance coverage [19]. This is higher than in low-income countries, where 7.9% of women have health insurance coverage [19]. However, lower than in upper-middle-income countries, 52.5% of women had health insurance coverage [19]. Since the formal launch of the Cambodia National Social Security Fund (NSSF) with the Health Insurance Scheme in 2008, the proportion of women with health insurance coverage has increased from 16% in 2014 to 22% in 2021–2022 [5]. This proportion exponentially increased due to the Royal Government of Cambodia's implementation of the NSSF for all workers in the formal and informal sectors of the economy [10]. Moreover, it has plans to extend the healthcare benefits under the NSSF to the family members of the employees as well [16].

~~The proportion of women with health insurance coverage was found to be highest among women with higher levels of education and those from better households. This is in line with a meta-analysis of 48 studies conducted in 17 countries, which revealed a higher likelihood of health insurance participation in the wealthiest and most educated households [21]. Additionally, women with higher educational levels and wealth status consistently predicted health insurance ownership among women at reproductive age across five Sub-Saharan African countries [22]. Furthermore, we fund women with health insurance coverage, a lower proportion among women living in rural areas. Consistent with studies in Nigeria [9]. This suggests that the more vulnerable populations, such as the poor, rural areas, and least educated, should be reached with government interventions such as having health insurance coverage.~~

~~The proportion of pregnant women attending four or more antenatal care appointments has increased considerably from 9% to 86.1% between 2000 and 2021.~~ This study found that women with health insurance coverage were 1.6 times more likely to attend four or more ANC visits during pregnancy. Previous studies documented the positive relationship between health insurance and the number of ANC visits among women of reproductive age [8,9,19]. Health insurance eliminates the financial barrier to accessing maternal health services caused by out-of-pocket payments. It has a beneficial effect in reducing the number of low-birth-weight babies born and child mortality [17,23]. The result is more equitable access to care, potentially improving maternal health outcomes [9,19]. The MoH has since raised the minimum standard for ANC visits during pregnancy to at least four trips [20,24]. The dramatically significant increase in the highest prevalence of four or more ANC visits was an effort by the Royal Government of Cambodia, which has strengthened health facilities across the country, particularly in rural areas, improved infrastructure, provided essential medical equipment and supplies, increased the number of midwives, expanded antenatal care, and provided more skilled medical practitioners at childbirth to ensure safe delivery practices. Furthermore, to encourage early and routine ANC visits, the government is offering pregnant women a monetary incentive of ~~US$~~20 US dollars for each visit during a maximum of four ANC visits at any health facility with a contract with the National Social Security Fund (NSSF) [14,25].

This study found that increased education and household wealth index increased the likelihood of four or more ANC visits. Women's education and higher wealth index are more likely to attend

four or more ANC visits. This aligns with previous evidence around socio-economic inequalities in maternal health service utilization in Cambodia and South Asia [26,27]. This may be because the more educated ~~the~~ wom~~e~~an is~~,~~ the more aware ~~she is~~ of the importance of ANC for her health and baby [28]. Additionally, education gives women the power to decide whether to seek medical attention and enables them to recognize warning signs of pregnancy complications. Moreover, ~~On the other hand,~~ women from higher-income households were more likely to be able to cover the costs of care-seeking, including any related expenses and transportation [26,27]. Thus, in this study, women with higher education levels and household wealth indexes had the highest proportion of health insurance coverage.

This study has several strengths. First, it used the most recent women's data from the 2021–2022 CDHS, an extensive representative national population-based household survey with a high response rate of 97%. Second, the recall bias has been minimized by limited analysis of women's most recent deliveries within the last two years preceding the survey [5]. ~~Data were collected using validated survey methods and highly trained data collectors, contributing to improved data quality [29].~~ Third, the complex survey design and sampling weights in the analysis accounted for both descriptive and analytics methods that enabled us to generalize our findings to the population of WRA in Cambodia. In addition, DHS data were collected using validated survey methods and highly trained data collectors, contributing to improved data quality [29]. Last~~ly~~, to our knowledge, this is the first study to report the association between health insurance coverage and ANC visits in Cambodia. After controlling sociodemographic factors, we found significant associations between health insurance coverage and several forums or more ANC visits. A key finding compares the association with other studies in Southeast Asia and globally.

Despite this, there are several limitations. First, this study ~~was~~ used a secondary analysis~~household based survey~~, so it did not address health institution factors of antenatal care utilization and service availability; hence, this study could not explore the quality of ANC services, though the quality of healthcare services plays a vital role in patient satisfaction and use. Second, the study's cross-sectional nature could not assist in the temporal relationship of variables including the number of years since women joined health insurance on ANC utilization. Therefore, further study should be conducted to identify factors related to health institutions. In addition, antenatal care utilization should be performed based on the new WHO guidelines revised in 2016 at the national level [24].

Moreover, longitudinal studies that address comprehensive variables should be studied. Third, we excluded other factors, such as maternal complications and women's empowerment indicators, that could affect the use of maternal care. Lastly, CDHS did not assess a direct measure of maternal health literacy.

Conclusion

This is the first study to report the association between health insurance coverage and ANC visits in the healthcare setting in Cambodia. Cambodian pregnant women attend four or more antenatal care visits, which is slightly high. However, it still needs to be satisfactory. Health among coverage women of reproductive age in Cambodia is relatively low. Moreover, we found that women with health insurance, women with education, and being rich in the wealth quintile were strong predictors of women attending four or more ANC visits. There is a need to pay close attention to improving the uptake of health insurance among women of reproductive age, especially targeting women with no education, from low-income families, and women who reside in rural areas. Policymakers may need to prioritize women of reproductive age in designing and implementing health insurance programs to increase their uptake. This would provide financial risk protection, facilitate access to maternal health services, and possible attainment of Cambodia's SDG 3 targets.

Acknowledgments

The authors would like to thank DHS-ICF, who approved the data used for this paper.

References

1. National Institute of Statistics, Directorate General for Health, and ICF International. Cambodia Demographic and Health Survey 2014. Phnom Penh, Cambodia, and Rockville, Maryland, USA: National Institute of Statistics, Directorate General for Health, and ICF International. Available online: https://dhsprogram.com/pubs/pdf/fr312/fr312.pdf. 2015.
2. National Institute of Statistics PP, Cambodia, Ministry of Health Phnom Penh, Cambodia, The DHS Program, ICF, Rockville, Maryland, USA. Cambodia Demographic and Health Survey 2021–22. Phnom Penh, Cambodia, and Rockville, Maryland, USA: NIS, MoH, and ICF. 2022. Available from: https://dhsprogram.com/pubs/pdf/FR377/FR377.pdf.
3. The United Nations Development Programme. The Sustainable Development Goals by 2016-2030. Available from: https://www.undp.org/content/undp/en/home/sustainable-development-goals.html.
4. Acuin CS, Khor GL, Liabsuetrakul T, Achadi EL, Htay TT, Firestone R, et al. Maternal, neonatal, and child health in southeast Asia: towards greater regional collaboration. Lancet. 2011;377(9764):516-25. Epub 20110125. doi: 10.1016/s0140-6736(10)62049-1. PubMed PMID: 21269675; PubMed Central PMCID: PMCPMC7159081.
5. National Institute of Statistics (NIS) MoHM, and ICF. . Cambodia Demographic and Health Survey 2021–22 Final Report. Phnom Penh, Cambodia, and Rockville, Maryland, USA: NIS, MoH, and ICF. 2023. Available from: https://dhsprogram.com/pubs/pdf/PR136/PR136.pdf.
6. Merga BT, Raru TB, Deressa A, Regassa LD, Gamachu M, Negash B, et al. The effect of health insurance coverage on antenatal care utilizations in Ethiopia: evidence from national survey. Front Health Serv. 2023;3:1101164. Epub 20231006. doi: 10.3389/frhs.2023.1101164. PubMed PMID: 37869683; PubMed Central PMCID: PMCPMC10587574.
7. Aboagye RG, Okyere J, Ahinkorah BO, Seidu AA, Zegeye B, Amu H, et al. Health insurance coverage and timely antenatal care attendance in sub-Saharan Africa. BMC Health Serv Res. 2022;22(1):181. Epub 20220211. doi: 10.1186/s12913-022-07601-6. PubMed PMID: 35148769; PubMed Central PMCID: PMCPMC8840787.
8. Wang W, Gheda Temsah, and Lindsay Mallick. . Health Insurance Coverage and Its Impact on Maternal Health Care Utilization in Low- and Middle-Income Countries. DHS Analytical Studies No. 45. Rockville, Maryland, USA: ICF International. . 2014.
9. Ahuru RR, Omon IJ, Nzoputam CI, Ekomoezor E. Health insurance ownership and maternal health service uptake among Nigerian women. HEALTH. 2021;12.
10. National Institute of Statistics. Ministry of Planning. General population census of the Kingdom of Cambodia 2019. Available from: https://www.nis.gov.kh/nis/Census2019/Final%20General%20Population%20Census%202019-English.pdf.
11. The World Bank In Cambodia. Overview 2019. Available from: https://www.worldbank.org/en/country/cambodia/overview.
12. World Health Organization (WHO). Global Health Expenditure Database 2019. Available from: https://apps.who.int/nha/database/PHC_Country_profile/Index/en.
13. World Health Organization (WHO); World Bank and WHO. Half the world lacks access to essential health services, 100 million still pushed into extreme poverty because of health expenses 2017. 2017. Available from: http://www.who.int/mediacentre/news/releases/2017/half-lacks-access/en/.
14. National Social Security Fund (NSSF). Health Insurance Scheme 2016. Available from: https://www.nssf.gov.kh/health-care-scheme-2/.
15. Nakamura H, Amimo F, Yi S, Tuot S, Yoshida T, Tobe M, et al. Implementing a sustainable health insurance system in Cambodia: a study protocol for developing and validating an efficient household income-level assessment model for equitable premium collection. Int J Equity Health. 2020;19(1):17. Epub 20200131. doi: 10.1186/s12939-020-1126-8. PubMed PMID: 32005237; PubMed Central PMCID: PMCPMC6995079.

16. The Royal Government of Cambodia. National Social Protection Policy Framework 2016–2025. Phnom Penh: The Council of Ministers; 2017.
17. Likka MH, Handalo DM, Weldsilase YA, Sinkie SO. The effect of community-based health insurance schemes on utilization of healthcare services in low- and middle-income countries: a systematic review protocol of quantitative evidence. JBI Database System Rev Implement Rep. 2018;16(3):653-61. doi: 10.11124/jbisrir-2017-003381. PubMed PMID: 29521866.
18. Dadjo J, Ahinkorah BO, Yaya S. Health insurance coverage and antenatal care services utilization in West Africa. BMC Health Serv Res. 2022;22(1):311. Epub 20220307. doi: 10.1186/s12913-022-07698-9. PubMed PMID: 35255895; PubMed Central PMCID: PMCPMC8899447.
19. Hooley B, Afriyie DO, Fink G, Tediosi F. Health insurance coverage in low-income and middle-income countries: progress made to date and related changes in private and public health expenditure. BMJ Glob Health. 2022;7(5). doi: 10.1136/bmjgh-2022-008722. PubMed PMID: 35537761; PubMed Central PMCID: PMCPMC9092126.
20. Ministry of Health. Guideline for implementation the service package of Antenatal Care Delivery and Post natal Care 2019 [updated 2019]. 3:[Available from: https://niph.org.kh/niph/uploads/library/pdf/GL220_NMCHC_anc_pnc_package_eng.pdf.
21. Osei Afriyie D, Krasniq B, Hooley B, Tediosi F, Fink G. Equity in health insurance schemes enrollment in low and middle-income countries: A systematic review and meta-analysis. Int J Equity Health. 2022;21(1):21. Epub 20220212. doi: 10.1186/s12939-021-01608-x. PubMed PMID: 35151323; PubMed Central PMCID: PMCPMC8841076.
22. Shao L, Wang Y, Wang X, Ji L, Huang R. Factors associated with health insurance ownership among women of reproductive age: A multicountry study in sub-Saharan Africa. PLoS One. 2022;17(4):e0264377. Epub 20220412. doi: 10.1371/journal.pone.0264377. PubMed PMID: 35413063; PubMed Central PMCID: PMCPMC9004737.
23. Imo CK, De Wet-Billings N, Isiugo-Abanihe UC. The impact of maternal health insurance coverage and adequate healthcare services utilisation on the risk of under-five mortality in Nigeria: a cross-sectional study. Arch Public Health. 2022;80(1):206. Epub 20220913. doi: 10.1186/s13690-022-00968-2. PubMed PMID: 36100949; PubMed Central PMCID: PMCPMC9472384.
24. WHO Guidelines Approved by the Guidelines Review Committee. WHO Recommendations on Antenatal Care for a Positive Pregnancy Experience. Geneva: World Health Organization

Copyright © World Health Organization 2016.; 2016.
25. Ministry of Information. Government Raises Allowances For Mothers And Children. Available from: https://www.information.gov.kh/articles/108509.
26. Chham S, Radovich E, Buffel V, Ir P, Wouters E. Determinants of the continuum of maternal health care in Cambodia: an analysis of the Cambodia demographic health survey 2014. BMC Pregnancy Childbirth. 2021;21(1):410. Epub 20210602. doi: 10.1186/s12884-021-03890-7. PubMed PMID: 34078318; PubMed Central PMCID: PMCPMC8170811.
27. Lee HY, Oh J, Kim R, Subramanian SV. Long-term trend in socioeconomic inequalities and geographic variation in the utilization of antenatal care service in India between 1998 and 2015. Health Serv Res. 2020;55(3):419-31. Epub 20200304. doi: 10.1111/1475-6773.13277. PubMed PMID: 32133652; PubMed Central PMCID: PMCPMC7240766.
28. Thakkar N, Alam P, Saxena D. Factors associated with underutilization of antenatal care in India: Results from 2019-2021 National Family Health Survey. PLoS One. 2023;18(5):e0285454. Epub 20230508. doi: 10.1371/journal.pone.0285454. PubMed PMID: 37155665; PubMed Central PMCID: PMCPMC10166529.
29. Allen C, Fleuret J, Ahmed J, editors. Data quality in demographic and health surveys that used long and short questionnaires2020: ICF.

Authors' point-by-point responses to the editor and reviewers

Manuscript ID: PGPH-D-24-00222

Manuscript Titled: Health insurance coverage and antenatal care utilization in Cambodia: Analysis of Cambodia Demographic and Health Survey 2021-22

PLOS Global Public Health

Dear editor and reviewers,

We would like to thank all the reviewers and editor for providing their important time and effort in reviewing our manuscript. We have addressed all comments where relevant, as listed below:

1. **Additional Editor Comments:**

This paper tried to examine the effects of health insurance coverage and antenatal care (ANC) utilization among pregnant women in Cambodia. Although the reviewers raised some concerns regarding this paper. My major concern is the analysis regarding the table 4 multiple logistic regression model where the authors showed a significant relationship among the insured and non-insured mother regarding the recommended ANC service utilization. The authors conclude compared to women without health insurance, those with health insurance coverage were 1.6 times more likely to attend four or more ANC visits. However, Table 4 also indicated that, most of the explanatory variables have a positive relationship with outcome variables. Even we observed that richest and higher educated mother utilized 3.2 and 3.1 times higher than their counterpart which is higher than having insurance variable. Therefore, I wondered education and wealth more important that having health insurances. Please clarify? The paper is fine, if the authors change the title of this paper to find out the determinants of ANC service utilization using Cambodia DHS data. However, to examine the effect of health insurance, I would like to see the reanalysis with two separate models for the mothers who were belonged to the health insurance coverage and who have not. Indeed, without propensity score matching, I believed the impact of health insurance on ANC service utilization may be questionable. See the related papers

Author's response: On page 1 of the manuscript, we have adopted and modified the title accordingly, and now reads "The effect of health insurance coverage on antenatal care utilization in Cambodia: A secondary analysis of Cambodia Demographic and Health Survey 2021-2022".

Author's response: We have revised the manuscript to explain the concerns with the result presented in "Table 4 multiple logistic regression model" To check for a high correlation among the independent variables, a test for multicollinearity was carried out among independent variables was checked before fitting the final multiple logistic regression model, including coverage health insurance, women's age,

number of children ever born, education, wealth index, occupation, marital status, and place of residence. The result of the evaluating variance inflation factor (VIF) scores after fitting an Ordinary Least Squares regression model with the mean value of VIF was 1.53, which is less than the cutoff point indicating no collinearity correlation among the independent variables. Next, a multiple logistic regression was conducted to examine the independent association between health insurance coverage and other covariates on ANC utilization. The selection of variables into the multiple logistic regression model was based on the previously published papers provided in reference numbers #6, #18 and their statistical significance in the Chi-square test at p-value ≤ 0.10.

Authors' response: We have asked professional editing services to revise and make corrections grammatically and accordingly, as suggested.

2. **Reviewers' comments:**

Reviewer #1: please correct 15-59 years as 15-49 years in line number 116 (page 4). Complete the line 133 -descriptive and analytical analysis or something like this. Make the line 141 correct, there is no verb in this sentence. Rewrite the sentence /line number149. Correct/rewrite line number 257,258. Read carefully the full text.

Authors' response: We have revised the manuscript accordingly.

Reviewer #2: Thank you for sharing the manuscript and would like to congratulate them for their nice work. My few comments/clarification questions are incorporated in the manuscript.

1. Did the study assess the cut-off period to identify the association between access to maternal health and years of membership/participation in health insurance?

   Authors' response: cut-off period or years of membership/participation in health insurance were not included in the CDHS 2022 questionnaire.

2. What is the particular interest of the researchers only using 2021-2022 data?

   Authors' response: The results are more reliable and reflect the current situation among women in Cambodia. Moreover, the findings of the study on the effect of health insurance coverage on antenatal care utilization are similar to other studies (for example, references #6, and #18), and the result can be used as a baseline value for comparison in the future.

3. How do you assess the confounding effects of other variables such as income, and level of education? Because those with better education and income are likely to attend 4 or more ANC visits.

   Authors' response:

The multicollinearity test was carried out among independent variables that were checked before fitting the final multiple logistic regression model, including coverage health insurance, women's age, number of children ever born, education, wealth index, occupation, marital status, and place of residence. The result of the evaluating variance inflation factor (VIF) scores after fitting an Ordinary Least Squares regression model with the mean value of VIF was 1.53, which is less than the cutoff point indicating no collinearity correlation among the independent variables.

Next, a multiple logistic regression was conducted to examine the independent association between health insurance coverage and other covariates on ANC utilization.

The selection of variables into the multiple logistic regression model was based on the previously published papers provided in reference numbers #6, #18 and their statistical significance in the Chi-square test at p-value ≤ 0.10.

For instance, according to multiple logistic regression models with an interaction term between wealth index and education, there is no association between several ANC visits and wealth and education.

4. Just for curiosity, is those women who did not attend any ANC visit categorized under less than 4 ANC visit?

Authors' response: less than 4 ANC visits were included in women who reported no ANC visits.

5. Did the study assess the association between the number of years since they joined the insurance and ANC use?

Authors' response: the number of years since they joined the health insurance was not included in the CDHS 2022 questionnaire. This is a limitation of the study.

Reviewer #3: Thank you for the opportunity to review this manuscript titled "Health insurance coverage and antenatal care utilization in Cambodia: Analysis of Cambodia Demographic and Health Survey 2021-22"

Title: I can see that title has two outcome variables (Health insurance coverage and ANC utilization) and no independent variable. It would be better to have this put to look a complete topic. Example; you can write, "Factors Associated with Health Insurance and ANC Utilization.........."

Author's response: We have modified the title accordingly, and now reads "The effect of health insurance coverage on antenatal care utilization in Cambodia: A secondary analysis of Cambodia Demographic and Health Survey 2021-2022".

Methods: In the outcome variable section you stated that "This study's outcome was the number of ANC visits..." I can see that you left out another important outcome variable, i.e Health

Insurance Coverage. Please update this section. Further, I can see that you used these two outcome variables very well in your result section (see Table 2 & Table 3).

Author's response: To clarify the outcome variable was women attended four or more ANC visits. Now, Table 2 removed.

Results: Update your topic that reflect the independent variables used in result. I gave a suggestion how the title can be improved.

Author's response: To clarify, now we have modified the title accordingly "The effect of health insurance coverage on antenatal care utilization in Cambodia: A secondary analysis of Cambodia Demographic and Health Survey 2021-2022". Table 2 removed.

Data sharing: Write a statement on data related to your study and indicate the URL link for the data

Author's response: We have revised the manuscript accordingly. *"The Demographic and Health Survey data are publicly available from the website: (URL: https://www.dhsprogram.com/data/dataset_admin) and are accessible after registration on the website. The authors did not have special access privileges."*

Reviewer #4: The manuscript adequately responds to the research question based on the predefined scope. This is a secondary analysis of data from a demographic and health survey. The author should be keen not to describe the survey in the methodology instead of the approaches of the current study. Minor comments are included in the attached manuscript for author review.

Author's response: We have revised the manuscript accordingly as following all reviewers suggested.

Reviewer #5: I am glad to have had the opportunity to review this pertinent and interesting paper. The paper addresses a relevant issue, both socially and scientifically; however, I believe some changes could clarify and improve the current manuscript.
Please find my comments below:

Author's response: We have revised the manuscript accordingly as following all reviewers suggested.
