# Supplementary material for: The effect of health insurance coverage on antenatal care utilization in Cambodia: A secondary analysis of Cambodia Demographic and Health Survey 2021–2022
Source: PLOS Glob Public Health. 2024 Nov 4;4(11):e0002954. doi: 10.1371/journal.pgph.0002954 (PMC11534221; doi:10.1371/journal.pgph.0002954)
Supplement: Attachment — Submitted filename: Response to reviewers.docx [file pgph.0002954.s003.docx]

**Authors’ point-by-point responses to the editor and reviewers**

Manuscript ID: PGPH-D-24-00222

Manuscript Titled: Health insurance coverage and antenatal care utilization in Cambodia: Analysis of Cambodia Demographic and Health Survey 2021-22

PLOS Global Public Health

**Dear editor and reviewers,**

We would like to thank all the reviewers and editor for providing their important time and effort in reviewing our manuscript. We have addressed all comments where relevant, as listed below:

1. **Additional Editor Comments:**

This paper tried to examine the effects of health insurance coverage and antenatal care (ANC) utilization among pregnant women in Cambodia. Although the reviewers raised some concerns regarding this paper. My major concern is the analysis regarding the table 4 multiple logistic regression model where the authors showed a significant relationship among the insured and non-insured mother regarding the recommended ANC service utilization. The authors conclude compared to women without health insurance, those with health insurance coverage were 1.6 times more likely to attend four or more ANC visits. However, Table 4 also indicated that, most of the explanatory variables have a positive relationship with outcome variables. Even we observed that richest and higher educated mother utilized 3.2 and 3.1 times higher than their counterpart which is higher than having insurance variable. Therefore, I wondered education and wealth more important that having health insurances. Please clarify? The paper is fine, if the authors change the title of this paper to find out the determinants of ANC service utilization using Cambodia DHS data. However, to examine the effect of health insurance, I would like to see the reanalysis with two separate models for the mothers who were belonged to the health insurance coverage and who have not. Indeed, without propensity score matching, I believed the impact of health insurance on ANC service utilization may be questionable. See the related papers

**Author's response**: On page 1 of the manuscript, we have adopted and modified the title accordingly, and now reads “The effect of health insurance coverage on antenatal care utilization in Cambodia: A secondary analysis of Cambodia Demographic and Health Survey 2021-2022”.

**Author's response**: We have revised the manuscript to explain the concerns with the result presented in “Table 4 multiple logistic regression model” To check for a high correlation among the independent variables, a test for multicollinearity was carried out among independent variables was checked before fitting the final multiple logistic regression model, including coverage health insurance, women's age, number of children ever born, education, wealth index, occupation, marital status, and place of residence. The result of the evaluating variance inflation factor (VIF) scores after fitting an Ordinary Least Squares regression model with the mean value of VIF was 1.53, which is less than the cutoff point indicating no collinearity correlation among the independent variables. Next, a multiple logistic regression was conducted to examine the independent association between health insurance coverage and other covariates on ANC utilization. The selection of variables into the multiple logistic regression model was based on the previously published papers provided in reference numbers #6, #18 and their statistical significance in the Chi-square test at p-value ≤ 0.10.

**Authors’ response:** We have asked professional editing services to revise and make corrections grammatically and accordingly, as suggested.

2. **Reviewers' comments:**

**Reviewer #1:** please correct 15-59 years as 15-49 years in line number 116 (page 4). Complete the line 133 -descriptive and analytical analysis or something like this. Make the line 141 correct, there is no verb in this sentence. Rewrite the sentence /line number149. Correct/rewrite line number 257,258. Read carefully the full text.

**Authors’ response:** We have revised the manuscript accordingly.

**Reviewer #2:** Thank you for sharing the manuscript and would like to congratulate them for their nice work. My few comments/clarification questions are incorporated in the manuscript.

1. Did the study assess the cut-off period to identify the association between access to maternal health and years of membership/participation in health insurance?

**Authors’ response:** cut-off period or years of membership/participation in health insurance were not included in the CDHS 2022 questionnaire.

2. What is the particular interest of the researchers only using 2021-2022 data?

**Authors’ response:** The results are more reliable and reflect the current situation among women in Cambodia. Moreover, the findings of the study on the effect of health insurance coverage on antenatal care utilization are similar to other studies (for example, references #6, and #18), and the result can be used as a baseline value for comparison in the future.

3. How do you assess the confounding effects of other variables such as income, and level of education? Because those with better education and income are likely to attend 4 or more ANC visits.

**Authors’ response:**

The multicollinearity test was carried out among independent variables that were checked before fitting the final multiple logistic regression model, including coverage health insurance, women's age, number of children ever born, education, wealth index, occupation, marital status, and place of residence. The result of the evaluating variance inflation factor (VIF) scores after fitting an Ordinary Least Squares regression model with the mean value of VIF was 1.53, which is less than the cutoff point indicating no collinearity correlation among the independent variables.

Next, a multiple logistic regression was conducted to examine the independent association between health insurance coverage and other covariates on ANC utilization.

The selection of variables into the multiple logistic regression model was based on the previously published papers provided in reference numbers #6, #18 and their statistical significance in the Chi-square test at p-value ≤ 0.10.

For instance, according to multiple logistic regression models with an interaction term between wealth index and education, there is no association between several ANC visits and wealth and education.

4. Just for curiosity, is those women who did not attend any ANC visit categorized under less than 4 ANC visit?

**Authors’ response:** less than 4 ANC visits were included in women who reported no ANC visits.

5. Did the study assess the association between the number of years since they joined the insurance and ANC use?

**Authors’ response:** the number of years since they joined the health insurance was not included in the CDHS 2022 questionnaire. This is a limitation of the study.

**Reviewer #3**: Thank you for the opportunity to review this manuscript titled "Health insurance coverage and antenatal care utilization in Cambodia: Analysis of Cambodia Demographic and Health Survey 2021-22"

Title: I can see that title has two outcome variables (Health insurance coverage and ANC utilization) and no independent variable. It would be better to have this put to look a complete topic. Example; you can write, "Factors Associated with Health Insurance and ANC Utilization..........."

**Author's response**: We have modified the title accordingly, and now reads “The effect of health insurance coverage on antenatal care utilization in Cambodia: A secondary analysis of Cambodia Demographic and Health Survey 2021-2022”.

Methods: In the outcome variable section you stated that "This study's outcome was the number of ANC visits..." I can see that you left out another important outcome variable, i.e Health Insurance Coverage. Please update this section. Further, I can see that you used these two outcome variables very well in your result section (see Table 2 & Table 3).

**Author's response**: To clarify the outcome variable was women attended four or more ANC visits. Now, Table 2 removed.

Results: Update your topic that reflect the independent variables used in result. I gave a suggestion how the title can be improved.

**Author's response**: To clarify, now we have modified the title accordingly “The effect of health insurance coverage on antenatal care utilization in Cambodia: A secondary analysis of Cambodia Demographic and Health Survey 2021-2022”. Table 2 removed.

Data sharing: Write a statement on data related to your study and indicate the URL link for the data

**Author's response**: We have revised the manuscript accordingly. *“The Demographic and Health Survey data are publicly available from the website: (URL:* [*https://www.dhsprogram.com/data/dataset_admin*](https://www.dhsprogram.com/data/dataset_admin)*) and are accessible after registration on the website. The authors did not have special access privileges.”*

**Reviewer #4:** The manuscript adequately responds to the research question based on the predefined scope. This is a secondary analysis of data from a demographic and health survey. The author should be keen not to describe the survey in the methodology instead of the approaches of the current study. Minor comments are included in the attached manuscript for author review.

**Author's response**: We have revised the manuscript accordingly as following all reviewers suggested.

**Reviewer #5:** I am glad to have had the opportunity to review this pertinent and interesting paper. The paper addresses a relevant issue, both socially and scientifically; however, I believe some changes could clarify and improve the current manuscript.
Please find my comments below:

**Author's response**: We have revised the manuscript accordingly as following all reviewers suggested.
