# Supplementary material for: The effect of health insurance coverage on antenatal care utilization in Cambodia: A secondary analysis of Cambodia Demographic and Health Survey 2021–2022
Source: PLOS Glob Public Health. 2024 Nov 4;4(11):e0002954. doi: 10.1371/journal.pgph.0002954 (PMC11534221; doi:10.1371/journal.pgph.0002954)
Supplement: Attachment — Submitted filename: Response to reviewers.docx [file pgph.0002954.s005.docx]

**Authors’ point-by-point responses to the editor and reviewers**

**Manuscript ID: PGPH-D-24-00222R1**

Manuscript Titled: The effect of health insurance coverage on antenatal care utilization in Cambodia: A secondary analysis of Cambodia Demographic and Health Survey 2021-2022

*PLOS Global Public Health*

**Dear Editor and reviewers,**

We thank all the reviewers and editors for their important time and effort in reviewing our manuscript. We have addressed all comments where relevant, as listed below:

**Journal Requirements:**

**1**. Please review your reference list to ensure that it is complete and correct. If you have cited papers that have been retracted, please include the rationale for doing so in the manuscript text, or remove these references and replace them with relevant current references. Any changes to the reference list should be mentioned in the rebuttal letter that accompanies your revised manuscript. If you need to cite a retracted article, indicate the article’s retracted status in the References list and also include a citation and full reference for the retraction notice.

**Author's response**: We ensure that all reference lists are complete and accurate.

**Additional Editor Comments (if provided):**
There is a great deal of room for improvement in both writing and grammar. I advise the authors to work with a writing coach or copy editor to improve the flow and readability of the text.

**Author's response**: We acknowledge that all reviewers, particularly Reviewer #4, have suggested improvements to align the manuscript with scientific writing norms and grammar practices.

**Reviewers' comments:**

**2. Review Comments to the Author**

**Reviewer #4:** The authors have fairly attempted to address the previous comments. However, the manuscript still needs a thorough proof reading. More suggestions can be found in the attachment.

**Author's response**: We acknowledge Reviewer #4 has suggested improvements to align the manuscript with scientific writing norms and grammar practices. In response, we have revised the manuscript accordingly, following all your suggestions in the attachment.

**Abstract**

- p.1 line 18: insert "have" before the word examined

**Author's response**: We have modified the objective on page 1, line 18 of the abstract accordingly, and it now reads, “*Therefore, this study has examined the effect of health insurance coverage on ANC utilization in Cambodia*”.

- p.1 line 19: suggested delete "was" from objective

**Author's response**: We have modified the objective on page 1, line 18 of the abstract accordingly, and it now reads, “*Therefore, this study has examined the effect of health insurance coverage on ANC utilization in Cambodia”.*

- p.1 line 22: We talk about the association between X "and" Y. Not "on"

**Author's response**: We have revised on page 1, line 22 of the abstract accordingly, and it now reads, “*Multiple logistic regression analysis was explored to assess the association between health insurance coverage with women who attended four or more ANC visits*”

- p.1 line 31: Check grammar. You may be deleting what sounds correct

**Author's response**: We have revised it accordingly.

- p.1 line 37: Insertion does not sound correct

**Author's response**: We have revised it accordingly.

**Introduction**

- p.3 line 82-83: Check for grammar? …; results indicate that women with health insurance coverage had higher odds attended four or more ANC visits than those without…..

**Author's response**: We have revised it accordingly, and it now reads, “…..*The results indicate that women with health insurance coverage had higher odds of attending four or more ANC visits than those without health insurance coverage*”….

- p.3 line 86-87: ...were more likely to attend four or more ANC visits

**Author's response**: We have revised it accordingly, and it now reads, *“…were more likely to attend four or more ANC visits”*

- p.3 line 89: choose one; the abbreviation is already defined in the earlier sections “..antenatal care (ANC)..”

**Author's response**: We have revised it accordingly for consistency abbreviation *“…ANC…”*

- p.5 line 132: … included women who reported no ANC visits and 1 = four or and more ANC visits…
