# Supplementary material for: The effect of health insurance coverage on antenatal care utilization in Cambodia: A secondary analysis of Cambodia Demographic and Health Survey 2021–2022
Source: PLOS Glob Public Health. 2024 Nov 4;4(11):e0002954. doi: 10.1371/journal.pgph.0002954 (PMC11534221; doi:10.1371/journal.pgph.0002954)
Supplement: Attachment — Submitted filename: Response to reviewers_R3.docx [file pgph.0002954.s006.docx]

**Authors’ point-by-point responses to the editor and reviewers**

PGPH-D-24-00222R2

The effect of health insurance coverage on antenatal care utilization in Cambodia: A secondary analysis of Cambodia Demographic and Health Survey 2021-2022

PLOS Global Public Health

Dear Dr. Um,

Thank you for submitting your manuscript to PLOS Global Public Health. After careful consideration, we feel that it has merit but does not fully meet PLOS Global Public Health’s publication criteria as it currently stands. Therefore, we invite you to submit a revised version of the manuscript that addresses the points raised during the review process.

Please submit your revised manuscript by Oct 27 2024 11:59PM. If you will need more time than this to complete your revisions, please reply to this message or contact the journal office at [globalpubhealth@plos.org](mailto:globalpubhealth@plos.org). When you're ready to submit your revision, log on to <https://www.editorialmanager.com/pgph/> and select the 'Submissions Needing Revision' folder to locate your manuscript file.

Please include the following items when submitting your revised manuscript:

A rebuttal letter that responds to each point raised by the editor and reviewer(s). You should upload this letter as a separate file labeled 'Response to Reviewers'.

A marked-up copy of your manuscript that highlights changes made to the original version. You should upload this as a separate file labeled 'Revised Manuscript with Track Changes'.

An unmarked version of your revised paper without tracked changes. You should upload this as a separate file labeled 'Manuscript'.

Guidelines for resubmitting your figure files are available below the reviewer comments at the end of this letter.

We look forward to receiving your revised manuscript.

Kind regards,

Abdur Razzaque Sarker
Academic Editor
PLOS Global Public Health

Journal Requirements:

Please review your reference list to ensure that it is complete and correct. If you have cited papers that have been retracted, please include the rationale for doing so in the manuscript text, or remove these references and replace them with relevant current references. Any changes to the reference list should be mentioned in the rebuttal letter that accompanies your revised manuscript. If you need to cite a retracted article, indicate the article’s retracted status in the References list and also include a citation and full reference for the retraction notice.

**Author's response**: We ensure that all reference lists are complete and accurate.

Additional Editor Comments (if provided):

There are still some typo errors. I would like to suggest a thorough copy edit of the revised version.

Author's response: We acknowledge and thank Mr. **Sopheap Suong**, Flinders University, Adelaide, South Australia, Australia, for helpful, professional proofreading.

[Note: HTML markup is below. Please do not edit.]

Reviewers' comments:

Author's response: We would like to thank all reviewers.
